# The DNA dioxygenase Tet1 regulates H3K27 modification and embryonic stem cell biology independent of its catalytic activity

**DOI:** 10.1093/nar/gkac089

**Published:** 2022-02-12

**Authors:** Stephanie Chrysanthou, Qin Tang, Joun Lee, Samuel J Taylor, Yilin Zhao, Ulrich Steidl, Deyou Zheng, Meelad M Dawlaty

**Affiliations:** Ruth L. and David S. Gottesman Institute for Stem Cell and Regenerative Medicine Research, Albert Einstein College of Medicine, 1301 Morris Park Ave, Bronx, NY 10461, USA; Department of Genetics, Albert Einstein College of Medicine, 1301 Morris Park Ave, Bronx, NY 10461, USA; Department of Developmental & Molecular Biology, Albert Einstein College of Medicine, 1300 Morris Park Ave, Bronx, NY 10461, USA; Ruth L. and David S. Gottesman Institute for Stem Cell and Regenerative Medicine Research, Albert Einstein College of Medicine, 1301 Morris Park Ave, Bronx, NY 10461, USA; Department of Genetics, Albert Einstein College of Medicine, 1301 Morris Park Ave, Bronx, NY 10461, USA; Department of Developmental & Molecular Biology, Albert Einstein College of Medicine, 1300 Morris Park Ave, Bronx, NY 10461, USA; Ruth L. and David S. Gottesman Institute for Stem Cell and Regenerative Medicine Research, Albert Einstein College of Medicine, 1301 Morris Park Ave, Bronx, NY 10461, USA; Department of Genetics, Albert Einstein College of Medicine, 1301 Morris Park Ave, Bronx, NY 10461, USA; Department of Developmental & Molecular Biology, Albert Einstein College of Medicine, 1300 Morris Park Ave, Bronx, NY 10461, USA; Ruth L. and David S. Gottesman Institute for Stem Cell and Regenerative Medicine Research, Albert Einstein College of Medicine, 1301 Morris Park Ave, Bronx, NY 10461, USA; Department of Cell Biology, Albert Einstein College of Medicine, 1300 Morris Park Ave, Bronx, NY, 10461, USA; Department of Medicine, Albert Einstein College of Medicine, 1300 Morris Park Ave, Bronx, NY, 10461, USA; Department of Genetics, Albert Einstein College of Medicine, 1301 Morris Park Ave, Bronx, NY 10461, USA; Ruth L. and David S. Gottesman Institute for Stem Cell and Regenerative Medicine Research, Albert Einstein College of Medicine, 1301 Morris Park Ave, Bronx, NY 10461, USA; Department of Cell Biology, Albert Einstein College of Medicine, 1300 Morris Park Ave, Bronx, NY, 10461, USA; Department of Medicine, Albert Einstein College of Medicine, 1300 Morris Park Ave, Bronx, NY, 10461, USA; Albert Einstein Cancer Center, Albert Einstein College of Medicine, 1300 Morris Park Ave, Bronx, NY 10461, USA; Department of Genetics, Albert Einstein College of Medicine, 1301 Morris Park Ave, Bronx, NY 10461, USA; Departments of Neurology and Neuroscience, Albert Einstein College of Medicine, 1300 Morris Park Ave, Bronx, NY 10461, USA; Ruth L. and David S. Gottesman Institute for Stem Cell and Regenerative Medicine Research, Albert Einstein College of Medicine, 1301 Morris Park Ave, Bronx, NY 10461, USA; Department of Genetics, Albert Einstein College of Medicine, 1301 Morris Park Ave, Bronx, NY 10461, USA; Department of Developmental & Molecular Biology, Albert Einstein College of Medicine, 1300 Morris Park Ave, Bronx, NY 10461, USA

## Abstract

Tet enzymes (Tet1/2/3) oxidize 5-methylcytosine to promote DNA demethylation and partner with chromatin modifiers to regulate gene expression. Tet1 is highly expressed in embryonic stem cells (ESCs), but its enzymatic and non-enzymatic roles in gene regulation are not dissected. We have generated Tet1 catalytically inactive (*Tet1^m/m^*) and knockout (*Tet1^−/−^*) ESCs and mice to study these functions. Loss of Tet1, but not loss of its catalytic activity, caused aberrant upregulation of bivalent (H3K4me3^+^; H3K27me3^+^) developmental genes, leading to defects in differentiation. Wild-type and catalytic-mutant Tet1 occupied similar genomic loci which overlapped with H3K27 tri-methyltransferase PRC2 and the deacetylase complex Sin3a at promoters of bivalent genes and with the helicase Chd4 at active genes. Loss of Tet1, but not loss of its catalytic activity, impaired enrichment of PRC2 and Sin3a at bivalent promoters leading to reduced H3K27 trimethylation and deacetylation, respectively, in absence of any changes in DNA methylation. *Tet1^−/−^*, but not *Tet1^m/m^*, embryos expressed higher levels of Gata6 and were developmentally delayed. Thus, the critical functions of Tet1 in ESCs and early development are mediated through its non-catalytic roles in regulating H3K27 modifications to silence developmental genes, and are more important than its catalytic functions in DNA demethylation.

## INTRODUCTION

Pluripotency, which is the ability of ESCs to differentiate to cell types of the three embryonic germ layers, is governed by epigenetic mechanisms of gene regulation (1). DNA methylation (i.e. addition of a methyl group to the 5′ carbon of cytosine, 5mC) is a major form of epigenetic modification in the eukaryotic genome (2). It is commonly seen in the context of CpG dinucleotides and canonically promotes transcriptional silencing during various biological processes including development (3). The DNA methyltransferase (DNMT) enzymes, which catalyze CpG methylation, are well-studied and the mechanisms of DNA demethylation involving the Ten eleven translocation (Tet) family of enzymes have emerged in the last decade (4–6). Tet enzymes are dioxygenases that catalyze the conversion of 5mC to 5-hydroxymethylcytosine (5hmC), 5-formlylcytosine (5fC) and 5-carboxylcytosine (5caC) (7–9). These modified bases can be removed from the genome by DNA glycosylases or DNA repair machineries resulting in active DNA demethylation (6). Alternatively, they can block access to the DNA maintenance methyltransferase DNMT1 during replication and promote passive demethylation (6). There are three Tet enzymes in mammals (Tet1, Tet2 and Tet3) and each has a conserved C-terminal domain responsible for its catalytic activity (7,10).

Tet enzymes are expressed in ESCs and during development to regulate critical biological processes (11) including ESC differentiation and lineage specification (12–14). Tet1 and Tet2 are expressed in ESCs and their levels decline upon differentiation concomitant with an increase in Tet3 expression (13,15). ESCs deficient for individual Tet enzymes have distinct differentiation defects *in vitro* while loss of all three *Tet* genes robustly impairs normal ESC lineage commitment (12–15). Mice lacking Tet1 are viable but smaller in size in 129/B6 background (12) or lethal in some other genetic backgrounds (16,17). Tet2 knockout mice are viable but develop myelodysplastic syndrome (MDS) and CMML-like disease by one year of age (18,19). Combined deficiency of Tet1 and Tet2 leads to some developmental defects and partial perinatal lethality (13). Embryos lacking all three Tet enzymes develop to blastocyst stage but cannot support post-implantation development due to failed gastrulation, underscoring the significance of Tet enzymes in embryogenesis (20).

While Tet enzymes are large multi-domain proteins, their catalytic activity is mainly confined to their C-terminus region (7,10). This domain alone can promote DNA hydroxylation both *in vitro* and *in vivo* (7,10). The functions of the non-catalytic regions of the protein are not well-established and are believed to be independent of enzyme activity. In addition to their roles in DNA oxidation and demethylation, some reports have associated Tet proteins in formation of chromatin regulatory complexes and recruitment of epigenetic modifiers to their targets (21–23). For example, all three Tet enzymes can form complexes with O-linked N-acetylglucosamine transferase (OGT) to facilitate protein O-GlcNAcylation including histone GlcNAcylation and gene activation (24,25). Of significance to ESC biology, Tet1 interacts with the Sin3a deacetylase complex to promote H3K27 deacetylation and partners with Polycomb repressive complex (PRC2) to mediate H3K27 trimethylation, thereby facilitating gene repression in ESCs (21,23). This is consistent with loss of Tet1 in ESC leading to aberrant gene activation. Likewise, in hematopoietic stem cells (HSCs) we have shown that loss of Tet2 vs. loss of its catalytic activity alone leads to distinct gene expression changes (19). While Tet2 knockout HSCs and mice exhibit differentiation defects in both myeloid and lymphoid lineages, loss of Tet2 catalytic activity only affects the myeloid lineage. These observations imply that the regulatory roles of Tet enzymes go beyond their canonical enzymatic activity in DNA hydroxylation and demethylation, and that they also contribute to gene regulation in stem cells in non-canonical fashions.

While the field has mainly investigated the enzymatic functions of Tet enzymes in ESCs as bona fide DNA demethylases, the biological significance of their non-enzymatic contributions are less studied. The present study addresses a key question in the field with respect to the regulation of ESCs by non-canonical roles of Tet1. Using a platform of Tet1 catalytic mutant and knockout ESCs and mice, we have dissected and defined the catalytic independent roles and requirements of Tet1 in ESC differentiation and development. We find that loss of Tet1, but not loss of its catalytic activity, leads to upregulation of bivalent (H3K4me3^+^; H3K27me3^+^) developmental genes and aberrant differentiation towards mesendoderm and trophectoderm lineages. We show that Tet1 facilitates recruitment of PRC2 (for H3K27 trimethylation) and Sin3a (for H3K27 deacetylation) to promoters of bivalent genes and the helicase Chd4 to promoters of active genes in a completely catalytic-independent fashion. While loss of Tet1 or loss of its catalytic activity increases global DNA methylation, this increase does not correlate with deregulation of bivalent genes. These findings have implications in development where Tet1 knockout mid-gestation embryos, in contrast to their Tet1 catalytic-deficient counterparts, are smaller in size and developmentally delayed, and show aberrant expression of mesendoderm markers during early embryogenesis. This study establishes that the bulk of the biologically critical regulatory functions of Tet1 in ESC differentiation and early development are primarily mediated through its non-catalytic roles in establishment of bivalency at developmental genes and preventing their untimely activation.

## MATERIALS AND METHODS

### Generation of Tet1 catalytic mutant and knockout mouse ESCs

For generation of V6.5 Tet1 catalytic mutant mouse ESCs, a gene block carrying nucleotide substitutions in *Tet1* exon 10 to introduce point mutations H1621Y and D1623A (equivalent to amino acids H1652Y and D1654A in longer transcript) was synthesized (Supplementary Table S1) and cloned in a vector backbone with Sbf1 and Fse1 sites. This donor plasmid along with a pX330 plasmid expressing Cas9 and a gRNA against Tet1 exon 10 (Supplementary Table S1) were used for gene editing in V6.5 ESCs (129/B6 mixed) following our previously published protocols (26). Targeted clones were screened by RFLP using a unique PsiI restriction site introduced with silent mutations in *Tet1* mutant allele. Properly edited homozygote clones were verified by Sanger sequencing. For generation of V6.5 *Tet1* knockout ESCs, two pX330 plasmids expressing Cas9 and gRNAs flanking *Tet1* exon 4 (Supplementary Table S1) were used to delete exon 4 in V6.5 mESCs following our previously published protocols (26). Targeted clones were screened by PCR using primers flanking exon 4 (Supplementary Table S1) and properly targeted homozygous clones were verified by Sanger sequencing. Three independent clones of each genotype were used for experiments.

### Generation of endogenously Flag-tagged Tet1 ESCs

To generate Tet1*-*FLAG ESC lines, a guide RNA (Supplementary Table S1) targeting the stop codon of *Tet1* gene was cloned into pX330-GFP vector. 1.5 μg of vector along with 3.5 μg donor ssDNA carrying a 3X Flag tag sequence (27) (Supplementary Table S1) were transfected into *Tet1^+/+^* and *Tet1^m/m^* V6.5 ESC using X-tremeGENE 9 DNA transfection reagent (Roche, 06365787001), following the manufacturer's instructions. GFP-positive cells were then sorted by flow cytometry 48 hr post transfection. 2.5 × 10^4^ single cells were plated on 10-cm dishes on feeders, and after one week of culture individual colonies were picked, expanded, and screened by PCR using primers outside the homology arms of the donor template, to confirm the modification of the endogenous locus. PCR products from homozygous *Tet1^+/+;Flag/Flag^* and *Tet1^m/m;Flag/Flag^* clones were Sanger sequenced to confirm that the 3xFlag tag was inserted in frame in the C-terminus of the *Tet1* coding sequence.

### Generation of *Tet1* catalytic mutant and *Tet1^f/f^;R26^CreER^* mice

A V6.5 *Tet1^+/m^* clone (129/B6) was injected into mouse blastocysts at Einstein transgenic core to obtain chimeric mice. Male chimeras were bred to C57/B6 female mice to obtain germ line *Tet1^+/^*^m^ mice which were further intercrossed to generate *Tet1^m/m^* mice in a mixed 129/B6 background. Our *Tet1^−/−^* mice, published previously (12), were also on a mixed 129/B6 background. To avoid any variations due to subtle background differences, *Tet1^−/−^* and *Tet1^m/m^* mice were intercrossed and subsequent animals were used to generate *Tet1^−/−^* and *Tet1^m/m^* embryos and mice for experiments. To generate *Tet1^f/f^^;^R26^CreER^* mice, we crossed our published *Tet1^f/f^* mice (129/B6) (28) with *Rosa26^CreER^* mice (Jax stock 004847).

### Cell culture and differentiation assays

All ESC lines were cultured on feeders in ESC media containing serum/LIF as described before (12,13). For RNA and DNA extractions, ESCs were pre-plated to remove feeders and then seeded on gelatin overnight before harvest. For embryoid body (EB) formation assays, pre-plated ESCs were seeded in ESC media without LIF in hanging drops for 2 days followed by culture on non-adherent plastic surface for 4 days. EBs were harvested on day 6 for analyses. Inhibitor treatments were as follows: Feeder-free ESCs on gelatin were treated with 1–2uM GSK-126 for 48 hr and 0.5–1uM GSK-J4 for 48 h.

### Teratoma formation assay

About 1 × 10^6^ mouse ESCs were injected subcutaneously into SCID mice (Taconic) as previously described (12). Mice were euthanized after 6 weeks, tumors were harvested and fixed in formalin for 2 days, embedded in paraffin blocks and sectioned. Sections were stained with hematoxylin and eosin for histological analysis following standard procedures. Immunohistochemical staining for Plf (anti-Proliferin 1:100, R&D AF1623) was also performed using standard protocols.

### Chimera assay

*Tet1^+/+^*, *Tet1^m/m^* and *Tet1^−/−^* mESCs were transduced with a lentiviral FUW-GFP vector expressing constitutive GFP. Cells with high GFP expression were sorted. Lines with strong and homogenous expression of GFP were established. GFP-labeled cells of each genotype were injected into blastocysts and surgically implanted into 2.5 d.p.c. pseudo-pregnant females at the Einstein Transgenic Core following standard procedures to generate chimeras. Pregnant females were sacrificed at E11.5 and embryos and placentas were harvested. Embryos were imaged using an inverted immunofluorescence scope. Embryos and placentas were subjected to trypsinization and dissociation to single cell suspension. Placenta suspensions were treated with RBC lysis buffer to lyse red blood cells and then washed twice with 1X PBS. Embryo and placenta cell suspensions were fixed with 70% ethanol and analyzed by flow cytometry to quantify % GFP-positive cells in each embryo and placenta using BD LSR II flow cytometer and FlowJo software. This quantitative approach allows for more accurate assessment of chimerism over the qualitative imaging approach where red blood cells in organs like placenta can lead to autofluorescence or labeled cells in deep layers of the organs cannot be detected.

### RT-qPCR

RNA was extracted from three independent ESC lines of each genotype (cultured on gelatin for 24 h) using Omega E.Z.N.A. Total RNA kit (R6834-02) and subjected to cDNA synthesis using SuperScript III kit (Invitrogen, 18080-400), according to manufacturer's guidelines. RT-qPCR was performed using SYBR green and primers shown in Supplementary Table S1 in a BD Applied Biosystems StepOne™ Real-Time PCR System following standard protocols. Data was normalized to *Gapdh*.

### Western blots

Western blots were performed as before (29,30). Briefly, cells were lysed in RIPA buffer (50 mM Tris–HCl, pH 7.4, 250 mM NaCl, 2% Nonidet-P40, 2.5 mM EDTA, 0.1% SDS, 0.5% DOC) supplemented with Halt PIC (Thermo, 78430) and PMSF (Sigma, 93482), lysates were quantified by BSA assay and resolved on 7–9% SDS-PAGE gel (Mini-PROTEAN electrophoresis chamber, Bio-Rad), and transferred to PVDF membranes (Mini Trans-Blot apparatus, Bio-Rad) in 5-10% methanol transfer buffer following manufacturer's protocols. Membranes were blocked in 5% milk in PBS with 0.1% Tween-20 (PBS-T) and incubated O/N at 4°C with primary antibodies (anti-Tet1 1:3000 (GeneTex, GTX125888), anti-Tet2 1:750 (Abcam, ab124297), anti-Lamin-b1 1:1000 (ABclonal, A1910), anti-Sin3a 1:1000 (Abcam, ab3479), anti-Ezh2 1:1000 (CST, 5246), anti-Chd4 1:1000 (Abcam, ab70469)). Secondary antibody incubations (HRP-anti-mouse, 401253, or HRP-anti-rabbit, 401393, Calbiochem 1:2500) were for 1 h at RT. Protein bands were identified with ECL chemiluminescence reagent (Amersham RPN2106). Lamin-b1 was used as loading control.

### Immunostaining of blastocysts and ESCs

Blastocysts were washed with PBS and fixed with 4% formaldehyde for 15 min at room temperature and then permeabilized with 0.5% Triton X-100 in PBS for 15 min and blocked in 3% BSA, 0.01% Tween-20 in PBS for 1 h at room temperature. They were incubated in primary antibody (anti-Gata6 1:300 (R&D, AF1700)) at 4°C overnight, and in secondary antibody (Alexa Flour 568-anti-goat, 1:500 (Thermo Fisher, A-11057)) at room temperature for an hour. Nuclei were stained with DAPI (1:1000, 5 ug/ml stock). Microscopy was performed using Leica SP8 Confocal Microscope (1S10OD023591-01) and images were analyzed by Volocity 3D image analysis software. ESCs were cultured on gelatin-coated coverslips and immunostaining was performed as explained above using anti-Oct3/4 (1:100, Santa Cruz, SC5279), anti-Sox2 (1:100, CST, 2748S), anti-Nanog (1:100, Bethyl Laboratories, A300-397A), anti-Esrrb (1:200, ProteinTech, 22644-1-AP) and imaged using an inverted fluorescence microscope.

### 5hmC quantification

DNA was isolated from feeder-free ESC (*n* = 3 of each) and analyzed by LC/MS at Einstein Mass Spectrometry core following published methods (31). Briefly, 2 μg of DNA was digested (37°C, >1 h) with a cocktail of nuclease enzymes (DNA Degradase Plus™, Zymo Research; 2.5 μl 10X DNA Degradase Reaction buffer, 1 μl DNA Degradase Plus) and upon addition of aqueous formic acid (25 μl, 0.1% v/v; final concentration 40 ng digested DNA/μl) was injected onto a reverse phase UPLC column (Eclipse C18 2.1 × 50 mm, 1.8 particle size, Agilent). Peak areas for dC, 5mdC and 5hmdC were measured using Agilent Mass Hunter Quantitative Analysis version B.06.00. 5hmC levels were quantified as % of all Cs.

### Co-immunoprecipitation

Co-immunoprecipitation experiments were performed as described before (29,30). Briefly, ESCs were washed and collected in ice-cold PBS supplemented with Halt PIC (Thermo 78430). Nuclear extracts were prepared and treated with benzonase nuclease (Sigma, E1014), and incubated with 4 μg of antibody (anti-Tet1 (GeneTex, GTX125888), Rabbit-IgG (CST, 3900) crosslinked to Protein G-conjugated magnetic beads (Dynabeads protein G, Invitrogen) overnight at 4°C. IgG was used as control. Immunocomplexes were washed with buffer containing 20 mM HEPES, pH 7.6, 10% glycerol, 100 mM KCl, 1.5 mM MgCl_2_, 0.2 mM EDTA. The proteins were eluted in 2× Laemlli buffer at 95°C and analyzed by western blot as described above.

### ChIP-qPCR

ChIP experiments were performed on three independent ESC lines following published protocols (32). Briefly, ESCs were cultured on gelatin, crosslinked with 1% formaldehyde, harvested in Farnham lysis buffer (5 mM PIPES pH8, 85 mM KCl, 0.5% NP-40, protease inhibitors), further lysed in RIPA buffer (1× PBS, 1% NP-40, 0.5% sodium deoxycholate, 0.1% SDS, protease inhibitors) and sonicated. 5 μg of primary antibody (anti-Ezh2 (CST, 5246), anti-Suz12 (CST, 3737), anti-Sin3a (Abcam, ab3479), anti-H3K27me3 (Millipore, 07449) were coupled to magnetic beads (Dynabeads M-280 Sheep Anti-Rabbit/Mouse IgG (Invitrogen 11203/1D) in PBS/BSA. A total of 500 μg sonicated chromatin was incubated with the bead-antibody complex for each ChIP at 4°C overnight. Beads containing immune-bound chromatin were washed in LiCl buffer (100 mM Tris pH 7.5, 500 mM LiCl, 1% NP-40, 1% sodium deoxycholate), in 1× TE buffer and eluted in IP elution buffer (1% SDS, 0.1 M NaHCO_3_). DNA was purified by QIAquick PCR purification kit (Qiagen 28104) and DNA concentration was measured using Qubit 2.0 Fluorometer (Invitrogen). Enrichment at specific loci was quantified by RT-qPCR using primers listed in Supplementary Table S1 as explained above. ChIP-qPCR signals were calculated as fold enrichment using IgG as control.

### Gene expression profiling by RNA-seq and data analysis

RNA was extracted from two independent ESC lines of each genotype (cultured on gelatin for 24 h) using Omega E.Z.N.A. Total RNA Kit I (R6834). RNA was barcoded and libraries were prepared. ERCC RNA spike in controls were included for data normalization. Libraries were subjected to 100 bp paired-end sequencing using Illumina NextSeq 500 platform at the Einstein Epigenomics core following their protocols. Adaptors and low quality bases were trimmed using Trim Galore (Version 0.3.7, https://www.bioinformatics.babraham.ac.uk/projects/trim_galore/). Trimmed reads were mapped to the mouse reference genome mm10 using TopHat2 (Version 2.0.13) (33). The gene annotation used for transcriptome alignment is RefSeq downloaded from the UCSC Table Browser (https://genome.ucsc.edu/cgi-bin/hgTables). HTseq (Version 0.11.0) (34) with option ‘–stranded = no’ was used to calculate read counts mapped to genes. The Fragments Per Kilobase of transcript per Million (FPKMs) were calculated using the Cufflinks package (Version 2.2.1) (35). Genes with mean FPKM > 1.0 in any group were retained for differential expression analysis using R package DESeq2 (36). *P*_adj_ < 0.05 was used for selecting differentially expressed genes (DEGs). Gene Ontology (GO) analysis was performed by DAVID (37). Heatmaps with k-means clustering were used to display gene expression pattern over groups or samples. Differentially expressed genes were compared to bivalent genes in ESCs (38), genes bound by Tet1 based on published Tet1 ChIP-seq data (GSE26833) (23), genes bound by Ezh2 based on published Ezh2 ChIP-seq data (GSM1014542) (39), genes bound by Sin3a based on published Sin3a ChIP-seq data (GSM611196) (21), and genes bound by Chd4 based on published Chd4 ChIP-seq data (GSM1499118) (40) in ESCs as presented in Supplementary Figure S4A.

### CUT&Tag and data analysis

CUT&Tag was applied to map the genome-wide distribution of Tet1, Ezh2, Sin3a and histone modifications in *Tet1^+/+^*, *Tet1^m/m^* and *Tet1^−/−^* mESC lines as previously described using one cell line of each genotype (41). Briefly, 3 × 10^5^ cells per cell line were harvested and lightly fixed with 0.5% formaldehyde. The cells were bound to Concavalin A-coated beads and incubated with the primary antibody (anti-FLAG-tag (CST, 2368), anti-Ezh2 (CST, 5246), anti-Sin3a (Abcam, ab3479), anti-Chd4 (CST, 12011), anti-H3K4me3 (Active Motif, 39060), anti-H3K27me3 (CST, 9733), anti-H3K27ac (Abcam, ab4729), IgG control (CST, 3900) at 4°C overnight. Antibodies are described in Supplementary Table S2. Samples were then incubated with a secondary antibody (guinea pig α-rabbit (Antibodies Online, ABIN101961)) followed by adding pre-loaded pA-Tn5 adapter complex (generated in house). Tagmentation buffer with magnesium ions was used to induce transposase fragmenting activity. DNA was extracted by phenol chloroform isoamyl alcohol and amplified by NEBNext HiFi 2x PCR Master mix. AMPure XP beads (#A63880) were used for post-PCR clean-up of the libraries. Libraries were subjected to 75 bp paired-end sequencing using Illumina NextSeq 500 platform at the Einstein Epigenomics core following their protocols. Trim Galore was used to remove low-quality reads and the paired-end reads were mapped to the mouse reference genome mm10 using bowtie2 (Version 2.2.3) (42) with options: –local –very-sensitive-local –no-unal –no-mixed –no-discordant -I 10 -X 700. Peak calling was performed using MACS2 (Version 2.1.0) (43) with options -f BAMPE –keep-dup all. To get clean peaks, both IgG and untagged wild type groups were used as control to remove backgrounds when calling peaks. For Ezh2, Sin3a and histone markers, only IgG was used as control to call peaks. To generate tracks data for IGV or genome browser visualization, module bamCoverage from deepTools (Version 3.1.0) (44) was used to calculate the coverage of reads per 50 bp bin, module bigwigCompare was used for control signal subtraction. Picard tools (Version 2.3.0, https://broadinstitute.github.io/picard/) was used to remove duplicated reads from each mapped file. To prepare data for enrichment plots and heatmaps, the samples were normalized to the same sequencing depth first and then computeMatrix from deepTools was used to calculate coverage scores per promoter-bound regions or Tet1-bound-bivalent-DEG-promoter regions by default parameters, plotProfile from deepTools was used to draw the average enrichment plot. Finally, to show signals or enrichment around peak (or promoter) regions, k-means ranked clustering normalization from seqMINER (v1.2.1) was used to create CUT&Tag read density data matrix and clusters. plotHeatmap from deepTools was used to draw enrichment plot and heatmap. Occupancy at ±2 kb of TSSs or peak centers was examined for enrichment at promoters or peaks, respectively. Peaks were annotated to different genomic regions by R package ‘ChIPseeker’ (Version 1.16.1) (45).

### CUT&RUN and data analysis

CUT&RUN was applied to map the genome-wide distribution of Chd4 in *Tet1^+/+^*, *Tet1^m/m^* and *Tet1^−/−^* mESC lines as previously described using one cell line of each genotype (46). Briefly, 5 × 10^5^ cells per cell line were harvested and bound to Concavalin A-coated beads. Bead-bound cells were incubated with primary antibody; anti-Chd4 (CST, 12011) and IgG control (CST, 3900) at 4°C overnight. The samples were then incubated with a secondary antibody (guinea pig α-rabbit (Antibodies Online, ABIN101961)) followed by incubation with pA-MNase (generated in house) and buffer with calcium ions to induce fragmenting activity at 0°C. The reaction was stopped by 2× STOP buffer (340 mM NaCl, 20 mM EDTA, 4 mM EGTA, 0.05% digitonin, 100 ug/ml RNaseA, 50 ug/ml glycogen) at 37°C for 30 min. The beads were discarded and DNA was extracted by phenol chloroform isoamyl alcohol. Libraries were prepared by the NEBNext Ultra II DNA Library Prep kit for Illumina (E7645) following manufacturer's guidelines. AMPure XP beads (#A63880) were used for post-PCR clean-up of the libraries. Libraries were subjected to 35 bp paired-end sequencing using Illumina NextSeq 500 platform at the Einstein Epigenomics core. Fastqc was used to assess data quality control and Trim Galore was used to remove low-quality reads and adapters. Then we followed the pipelines in CUT&RUNTools (47) for alignment, peak filtering and peak calling. Bowtie2 was used for genomic mapping with option ‘–dovetail’. After alignment, reads with fragments ≤120 bp were filtered out and used for peak calling. MACS2 was applied with the default setting. Methods of data normalization, enrichment plotting and heatmap plotting were the same as described in CUT&Tag above.

### WGBS and data analysis

WGBS was performed using two independent lines of each *Tet1^+/+^*, *Tet1^m/m^* and *Tet1^−/−^* v6.5 mouse ESCs. High quality DNA was extracted by the Quick-DNA Miniprep Kit (Zymo, D3024), according to manufacturer's guidelines. Bisulfite conversion and sequencing were carried out by BGI Genomics company (https://en.genomics.cn/). Lamda DNA spike-in was added to confirm the bisulfite conversion efficiency (99.4% C was successfully bisulfite converted). The libraries with DNA fragment length of 100–300 bp were subjected to 100 bp pair-end sequencing on Illumina HiSeq 4000 platform. After sequencing, the raw reads were filtered by SOAPnuke (Version 1.5.5) (https://github.com/BGI-flexlab/SOAPnuke) with the parameters -n 0.001 -l 20 -q 0.4 -A 0.25 -Q 2 -G to remove adaptor sequences, contamination and low-quality reads. Then the clean data were mapped to mouse genome mm10 by Bismark (Version 0.18.1) (48) with default options. Duplicated sequences were removed by deduplicate_bismark. Coverage depth was calculated by SAMtools and methylation level of each cytosine were extracted by Bismark function ‘bismark_methylation_extractor’ from bam files. Methylpy (Version 1.4.0) (49) was used to call methylation state for all Cs over the genome and convert the methylation level to bigwig files which can be visualized by IGV or similar browser. R package methimpute (Version 1.8.0) (50) was used to bin the genome into 100 bp-tiles and calculate methylated counts and total counts for each tile. Symmetric CpGs were extracted by MethPipe (Version 3.4.3) (51). Differentially methylated sites (DMSs) were identified from these symmetric CpGs using beta-binomial regression method of MethPipe by thresholds of FDR < 0.05 and methylation difference ≥20%. Differentially methylated regions (DMRs) were identified by R package ‘bsseq’ (Version 1.16.1) from BSmooth (52) function and filtered by threshold of methylation difference ≥10% for each region. Genome-wide methylation distribution was indicated by cumulative plot generated by R, tiles with at least one CpG site and one read counts were used for the plots. There were 27,255,203 100 bp-tiles generated from the genome in total, only 12,367,274 tiles remained after filtering according to at least one CpG site in a tile and read counts ≥1. For cumulative plot, two-sample Kolmogorov-Smirnov test was used to test the significant difference between two curves, the *D* statistic and *P* values were calculated using the ‘ks.test’ function as implemented in R. In the tests, the null hypothesis is that cumulative distribution function (CDF) of *Tet1^+/+^* lies below *Tet1^m/m^* and *Tet1^−/−^*, while the alternative hypothesis is that CDF of *Tet1^+/+^* lies above *Tet1^m/m^* and *Tet1^−/−^*. For *Tet1^−/−^* versus *Tet1^m/m^*, the null hypothesis is *Tet1^−/−^* lies above *Tet1^m/m^*, while the alternative hypothesis is that *Tet1^−/−^* lies below *Tet1^m/m^*. The annotation of DMRs to gene elements were implemented by R package ‘ChIPseeker’. Violin plot was used to indicate CpG methylation level at different regions of mouse genome. CpGs used for violin plot were filtered by coverage of at least 10 in any of *Tet1^+/+^*, *Tet1^m/m^* and *Tet1^−/−^* groups. In total, 38,336,128 sites were used for the plot. The average methylation profile was calculated for both 5 kb upstream and downstream from the center of each DMR in 50 bp bins and plotted by plotProfile function from deepTools. Methylation differences were calculated by subtracting average methylation in *Tet1^+/+^* from those in *Tet1^−/−^* or *Tet1^m/m^*. In all figures, the two biological replicates in each group were merged together to calculate the methylation level. To detect DNA methylation dynamics at Transposable Elements (TEs) in *Tet1^+/+^*, *Tet1^m/m^* and *Tet1^−/−^*ESCs, we focused on four TE subfamilies which are variable in methylation level during development (53). Their genomic positions were collected from Dfam database (https://dfam.org/home) and methylation levels around the center of TE subfamilies (±5 kb) was examined and shown by composite plot. Methylation canyons were identified by ‘HMR’ program from MethPipe software. The ‘HMR’ program uses hidden Markov model (HMM) approach to identify hypo-methylated regions (HMRs). Totally 48920 HMRs were identified from *Tet1^+/+^* ESCs. CpG methylation levels and Tet1 binding signals at these regions were visualized in the same plot. Motif analysis of common DMRs were performed by HOMER (Version 4.7).

### ATAC-seq and data analysis

ATAC-seq was performed on two independent *Tet1^+/+^*, *Tet1^m/m^* and *Tet1^−/−^* v6.5 mouse ESC lines. 5 × 10^4^ cells were lysed in 50 ul ATAC-Resuspension Buffer (RSB) (0.1% NP-40, 0.1% Tween-20, 0.01% Digitonin (G9441)). Cell lysates were washed in 1ml cold ATAC-RSB without NP-40 nor digitonin. Cell pellet was resuspended in 50 ul transposition mix (1x TD buffer (15027866), 100 nM transposase (Nextera Tn5 Transposase, 15027865), 0.01% digitonin, 0.1% Tween-20, 16.5 ul PBS, 5 ul H_2_O) and the reaction was incubated at 37°C for 30 min. The reaction was cleaned-up with Zymo DNA Clean and Concentrator-5 kit (D4014) following manufacturer's instructions. The transposed DNA fragments were amplified using Nextera DNA CD Indices (20018707) and NEBNext High-Fidelity 2× PCR Master Mix (M0541) following manufacturer's protocols. Libraries (200–1000 bp fragments) were purified by double-sided bead purification using AMPure XP beads (A63880). Library quality was assessed on an Agilent High Sensitivity DNA Bioanalysis chip and quantified by Qubit 2.0 Fluorometer (Invitrogen). Libraries were sequenced at the Einstein Epigenomics core with a 75 bp paired-end protocol on an Illumina NextSeq 500 platform. Raw reads were trimmed by Trim Galore to remove adaptors and then mapped against the mm10 mouse genome using Bowtie2 with the option ‘-X 2000 –no-mixed –no-discordant –local’. Unmapped reads, mitochondrial reads and duplicated reads were removed by SAMtools (Version 1.9) (54) and Picard. Correlation between biological replicates was assessed to ensure high reproducibility before pooling each set of replicates. MACS2 was run with the options ‘-f BAMPE -g mm –nomodel –nolambda –slocal 10000 –keep-dup all’ to call peaks. Peaks from individual samples in each group of *Tet1^+/+^*, *Tet1^m/m^* and *Tet1^−/−^* were merged with bedtools2 (Version 2.26.0) (55) using parameter ‘-d 10’, and mm10 blacklist regions were filtered. Read coverage of each ATAC-seq peak was calculated using HTseq with parameter ‘–nonunique all’. The differentially accessible regions were identified using DESeq2 with threshold *P*_adj_ <0.05. Peaks annotations were implemented by R package ‘ChIPseeker’.

### Embryo analysis

All studies were performed in accordance with our IACUC approved protocols overseen by the Institute for Animal Studies of Albert Einstein College of Medicine. Mice were maintained on a 12/12 h light/dark cycle. Adult 2- to 5-month-old *Tet1^+/m^* and *Tet1^+/−^* animals were time-mated to generate *Tet1^m/m^* and *Tet1^−/−^* embryos, respectively. Pregnant mice were sacrificed at selected gestation time points and embryos were harvested, imaged, weighed, somites counted, and then genotyped by PCR using embryo tail or yolk sac DNA. For inducible deletion of *Tet1* in embryos, male *Tet1^f/f^^;^R26^CreER^* mice were time-mated with female *Tet1^f/f^* mice. Pregnant mice were administered Tamoxifen (IP, 80 mg/kg) at E7.5 to induce *Tet1* deletion in embryos. Embryos were harvested at E11.5 and analyzed and genotyped. For blastocyst analyses, homozygous animals were time-mated and blastocysts were harvested by flushing out uteri at E3.5. Blastocysts were either collected for RNA extraction and cDNA prep by SMART-Seq v4 Ultra Low Input RNA kit (Takara Bio, 634895) following manufacturer's instructions, and subsequent RT-qPCR, or they were fixed in 4% formaldehyde for immunofluorescence staining as explained above.

### Quantification and statistical analyses

One-way-ANOVA test or unpaired t-test in GraphPad Prism 7 were used for calculating statistical significance in RT-qPCR, ChIP-qPCR and embryo analyses. Statistical methods for analysis of genome-wide data sets are explained in detail under the respective sections.

## RESULTS

### Generation of Tet1 catalytic mutant (*Tet1^m/m^*) and knockout (*Tet1^−/−^*) ESCs

**Figure 1.**
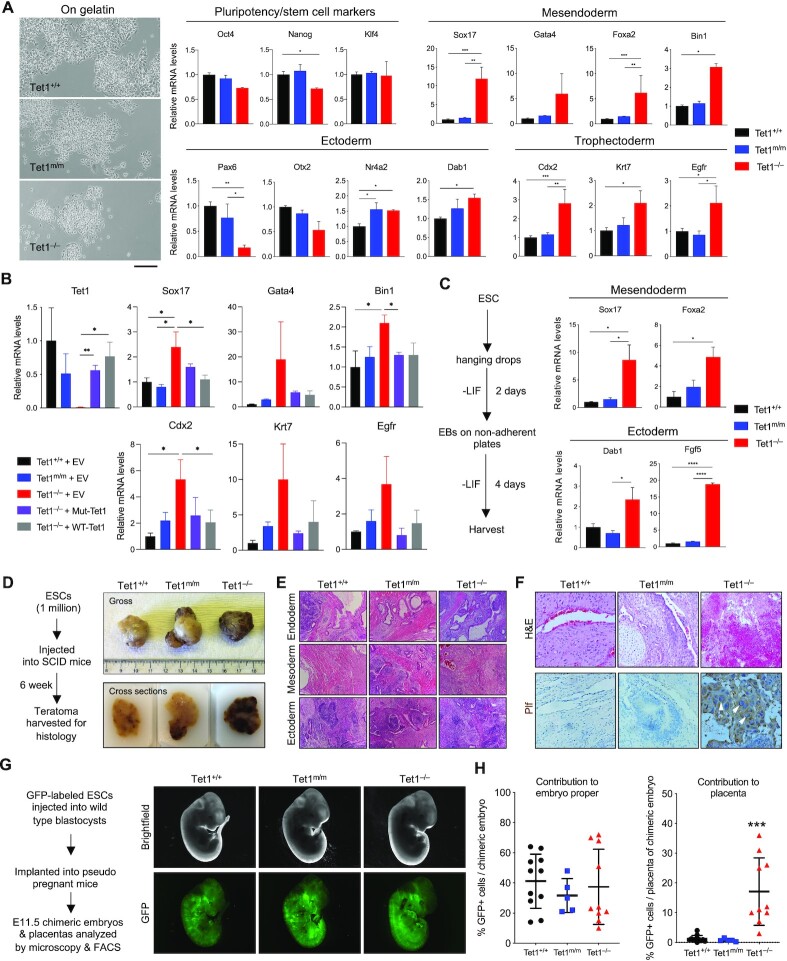
*Tet1^−/−^*, but not *Tet1^m/m^*, ESCs have aberrant differentiation toward mesendoderm and trophectoderm lineages. (**A**) Brightfield images of ESCs of indicated genotypes cultured on gelatin in ESC media (+serum/LIF) (left) and mRNA levels of pluripotency and lineage markers quantified by RT-qPCR (right). Data normalized to *Gapdh* expression. Three independent ESC lines of each genotype were used. One-way ANOVA test is used to assess statistical significance. Scale bar = 100um. (**B**) Quantification of lineage markers in *Tet1^+/+^* and *Tet1^m/m^* ESCs expressing an empty vector and *Tet1^−/−^* ESCs expressing wild type or catalytic mutant Tet1 or an empty vector by RT-qPCR. Data normalized to *Gapdh* expression. Two independent ESC lines of each genotype were used. One-way ANOVA test is used to assess statistical significance. (**C**) Schematic of Embryoid Body (EB) formation assay (left) and quantification of lineage marker mRNA levels in day 6 EBs by RT-qPCR (right). Data normalized to *Gapdh* expression. Three independent ESC lines of each genotype were used. One-way ANOVA test is used to assess statistical significance. (**D**) Schematic of the teratoma assay formation (left) and gross images of formalin fixed teratomas (top) and cross section images in paraffin blocks (bottom). Two replicates of each genotype used in this assay. (**E**) Hematoxylin and eosin (H&E) staining of teratoma sections confirming the presence of structures belonging to the three embryonic germ layers. (**F**) H&E staining of teratoma sections showing hemorrhage in *Tet1^−/−^* teratomas versus *Tet1^+/+^* and *Tet1^m/m^*. IHC staining for trophoblast giant cell marker Plf in teratomas of indicated genotypes. Arrows show enlarged nuclei representing trophoblast giant cells. (**G**) Gross and immunofluorescence images of chimeric E11.5 embryos generated by injection of GFP-labeled *Tet1^+/+^*, *Tet1^m/m^* or *Tet1^−/−^* ESCs into wild type E3.5 blastocysts. (**H**) Contribution of GFP-positive ESCs of indicated genotypes to the embryo proper (left) and placenta (right) quantified by flow cytometry and plotted. Note that ESCs of all genotypes contributed to the embryo proper (left), whereas only *Tet1^−/−^*, but not *Tet1^m/m^* or *Tet1^+/+^*, ESCs contributed to the placenta. One-way ANOVA test is used to assess statistical significance. In all panels error bars represent SEM. Statistically significant * *P* < 0.05, ** *P* < 0.01, *** *P* < 0.001.

To establish the enzymatic and non-enzymatic functions of Tet1 in ESCs, we generated ESC lines that lacked only the Tet1 catalytic activity (catalytic mutant or *Tet1^m/m^*) or the entire Tet1 protein (knockout or *Tet1^−/−^*) (*n* = 3 of each) (Supplementary Figure S1). For generation of *Tet1^m/m^* ESCs, we introduced point mutations in exon 10 of *Tet1* for amino acid substitutions H1621Y and D1623A (equivalent to amino acids H1652Y and D1654A in the longer transcript) in the iron binding pocket of Tet1 using CRISPR/Cas9-based gene editing in V6.5 mouse ESCs (Supplementary Figure S1A). These mutations are previously shown to render Tet enzymes completely catalytically-inactive without any dominant negative effects (7,10). For generation of *Tet1^−/−^* ESCs, we deleted exon 4 of *Tet1* using a pair of flanking gRNAs (Supplementary Figure S1A). Deletion of this exon, as previously shown (12), leads to complete loss of Tet1 protein. Genotypes of properly targeted ESCs were confirmed by PCR and RFLP (Supplementary Figure S1B) and verified by Sanger sequencing (Supplementary Figure S1C). Complete loss of Tet1 mRNA and protein in *Tet1^−/−^* ESCs as well as normal expression of Tet1 catalytic mutant mRNA and protein in *Tet1^m/m^* ESCs were confirmed by RTqPCR and Western blot, respectively (Supplementary Figure S1D and E). Consistent with loss of Tet1 catalytic activity in *Tet1^m/m^* ESCs, DNA isolated from *Tet1^m/m^* ESCs had ∼30% reduction in 5hmC levels measured by mass spectrometry, comparable to the levels present in *Tet1^−/−^* ESCs (Supplementary Figure S1F). All *Tet1^m/m^* and *Tet1^−/−^* ESC lines maintained normal ESC morphology when cultured on feeders or gelatin (Figure 1A and Supplementary Figure S2A). *Tet1^−/−^* ESCs had reduced clonogenicity compared to *Tet1^+/+^* and *Tet1^m/m^* ESCs when cultured on gelatin (Supplementary Figure S2B). However, this was not associated with loss of pluripotency, or rise of differentiated cells within *Tet1^−/−^* colonies, as pluripotency markers (Oct4, Sox2, Nanog and Esrrb) were comparably expressed in colonies of *Tet1^+/+^*, *Tet1^m/m^* and *Tet1^−/−^* ESCs (Supplementary Figure S2C). Since *Tet1^−/−^* ESCs lack all functions of the protein and the *Tet1^m/m^* ESCs only lack the enzymatic activity of the protein, these lines serve as a viable platform for dissecting the catalytic-dependent and independent functions of Tet1 in ESC biology.

### Loss of Tet1, but not loss of its catalytic activity, in ESCs leads to aberrant expression of lineage specifiers and compromised differentiation

To examine the effects of loss of Tet1 vs. loss of its catalytic activity on pluripotency of ESCs, we quantified the expression of selected pluripotency genes and several germ layer markers in *Tet1^−/−^*, *Tet1^m/m^* and wild type ESCs (Figure 1A). We found the expression of pluripotency factors to be unaffected with the exception of *Nanog* that was slightly downregulated in *Tet1^−/−^* ESCs. In contrast, we found robust upregulation of several mesendoderm (Sox17, Gata4, Foxa2, Bin1) and trophectoderm (Cdx2, Krt7, Egfr) markers in *Tet1^−/−^*, but not in *Tet1^m/m^* and wild type ESCs (Figure 1A). Interestingly, ectoderm marker Pax6 was significantly downregulated exclusively in *Tet1^−/−^* ESCs while some other markers like Nr4a2 were affected in both *Tet1^−/−^* and *Tet1^m/m^* ESCs. Overexpression of a catalytically dead *Tet1* transgene in *Tet1^−/−^* ESCs restored aberrant upregulation of several mesendoderm, trophectoderm markers to the levels present in *Tet1^+/+^*and *Tet1^m/m^* ESC, as effectively as overexpression of a *Tet1* wild type transgene (Figure 1B and Supplementary Figure S2D). These findings suggest that loss of Tet1, but not loss of its catalytic activity, leads to aberrant upregulation of mesendoderm and trophectoderm markers in ESCs. To examine if this promotes aberrant differentiation towards these lineages, we differentiated *Tet1^−/−^* and *Tet1^m/m^* ESCs to embryoid bodies (EBs). We found that *Tet1^−/−^* EBs expressed significantly increased mRNA levels of all three germ layer markers compared to *Tet1^m/m^* and wild type ESCs (Figure 1C). Together, these observations suggest that deficiency of Tet1, but not of its catalytic activity, compromises proper differentiation of ESCs.

### Tet1 knockout, but not catalytic mutant, ESCs form hemorrhagic teratomas and contribute to developing placenta in a chimera assay

To further investigate the catalytic and non-catalytic requirements of Tet1 in ESC pluripotency, we subjected *Tet1^−/−^*, *Tet1^m/m^* and wild type ESCs to a teratoma formation assay (Figure 1D). These lines formed teratomas with structures belonging to the three embryonic germ layers, suggesting that deficiency of Tet1 or mere loss of its enzymatic activity does not inhibit pluripotency of ESCs (Figure 1D and E). However, in a striking contrast, we found that *Tet1^−/−^* teratomas, consistent with previous studies (12,15) were excessively hemorrhagic, while *Tet1^m/m^* teratomas were not. Staining for the trophoblast giant cell marker Plf confirmed the increased presence of these cells in *Tet1^−/−^* teratomas (Figure 1F). These distinct features of *Tet1*^−/−^ and *Tet1^m/m^* teratomas are consistent with our earlier findings that loss of Tet1 but not loss of its catalytic activity causes abnormal activation of trophectoderm markers, and thereby leads to skewed differentiation towards this lineage. To examine if this defect in differentiation affects the developmental potential of ESCs during embryogenesis, we performed a chimera formation assay where we injected GFP-labeled *Tet1^+/^^+^* or *Tet1^m/m^* or *Tet1^−/−^* ESCs into wild type blastocysts and implanted them into recipient mice to form chimeric embryos (Figure 1G). Analysis of E11.5 embryos and placentas for presence of GFP-labeled cells revealed that while all three ESC lines contributed equally to the developing embryo proper, only *Tet1^−/−^*, but not *Tet1^+/^^+^* and *Tet1^m/m^*, ESCs contributed to placenta (Figure 1G and H). 8/10 *Tet1^−/−^* chimeric embryos had 10–40% GFP positive cells in their placentas compared to 0/5 *Tet1^m/m^* and 0/11 *Tet1^+/^^+^* embryos (Figure 1H). This is consistent with abnormal differentiation of *Tet1^−/−^* ESCs towards trophectoderm lineage. Together, these findings suggest that the non-catalytic functions of Tet1 suppress aberrant differentiation of ESCs towards the trophectoderm lineage.

### Loss of Tet1, but not loss of its catalytic activity, leads to aberrant activation of developmental genes in ESCs

**Figure 2.**
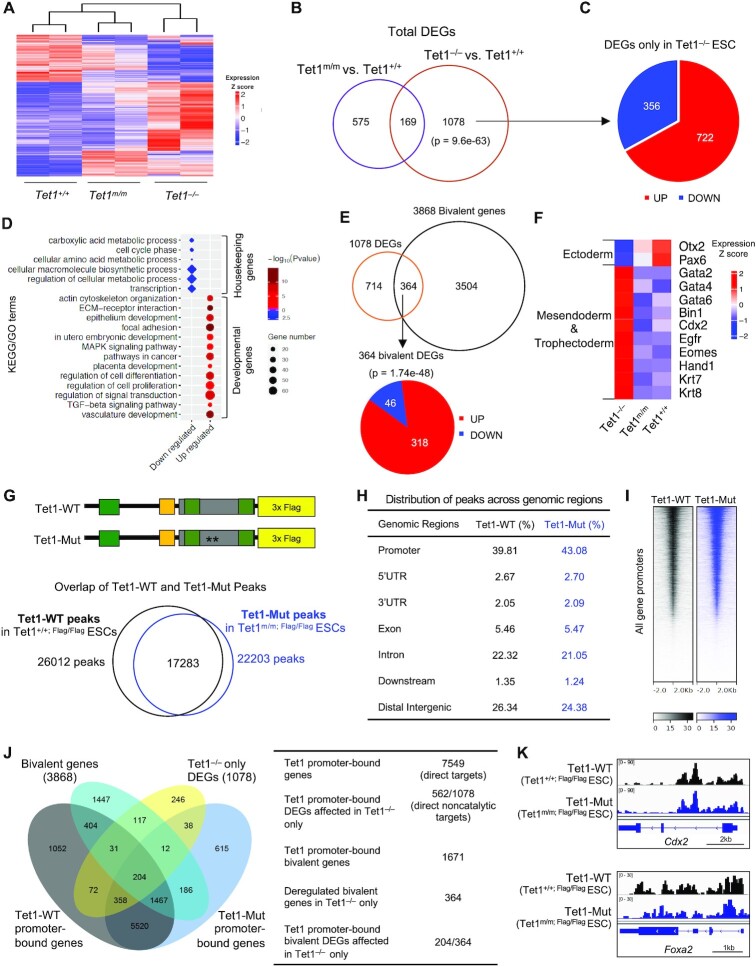
Aberrant activation of bivalent genes in *Tet1^−/−^*, but not in *Tet1^m/m^*, ESCs. (**A**) Heatmap of differentially expressed genes (DEGs) among *Tet1^+/+^*, *Tet1^m/m^* and *Tet1^−/−^* ESC lines (1822 DEGs in total). Colors indicate relative expression. Two independent ESC lines of each genotype were used. (**B**) Venn diagram showing overlap of differentially expressed genes between *Tet1^m/m^* and *Tet1^−/−^* ESCs when compared to *Tet1^+/+^* ESCs. Hypergeometric test was used to calculate p-values. (**C**) Distribution of genes uniquely upregulated (red) and downregulated (blue) in *Tet1^−/−^* ESCs. Note that the majority of differentially expressed genes are upregulated. (**D**) GO and KEGG pathway analysis of genes uniquely differentially expressed in *Tet1^−/−^* ESCs. (**E**) Venn diagram showing overlap between the 1078 uniquely deregulated genes in *Tet1^−/−^* ESCs and all 3868 bivalent genes in ESCs. Note that one-third of differentially expressed genes are bivalent and majority are upregulated. (**F**) Heatmap of expression of selected lineage specifier genes in indicated ESC lines. (**G**) Schematic of endogenously Flag-tagged wild type and catalytic mutant Tet1 protein in *Tet1^+/+,^^Flag/Flag^* and *Tet1^m/m, Flag/Flag^* ESCs respectively (top). Overlap of wild type and catalytic mutant Tet1 peaks as identified by CUT&Tag in *Tet1^+/+, Flag/Flag^* and *Tet1^m/m, Flag/Flag^* ESCs (bottom). (**H**) Distribution of wild type and catalytic mutant Tet1 peaks across genomic regions in *Tet1^+/+, Flag/Flag^* and *Tet1^m/m, Flag/Flag^* ESCs. (**I**) Heatmap showing CUT&Tag read densities for wild type and catalytic mutant Tet1 at all gene promoters (±2 kb of transcriptional start site (TSS)). (**J**) Overlap of Tet1 promoter-bound genes with bivalent genes and differentially expressed genes depicted in Venn diagram and summarized in table. (**K**) Genome browser track snapshots showing enrichment of wild type and catalytic mutant Tet1 CUT&Tag signals at selected developmental genes in *Tet1^+/+, Flag/Flag^* and *Tet1^m/m, Flag/Flag^* ESCs.

To gain molecular insights into the enzymatic and non-enzymatic functions of Tet1 in ESC gene expression programs, we compared the transcriptomes of *Tet1^−/−^*, *Tet1^m/m^* and wild type ESCs by RNA-seq (Figure 2A and Supplementary Figure S3A and Supplementary Table S3). To assess the global transcriptomic differences among the ESCs of the three genotypes and their replicates, we performed principal components analysis (PCA) which revealed that the biological replicates were clustered together but the three genotypes were well separated, with the first PC distinguishing *Tet1^+/+^* from *Tet1^−/−^* and *Tet1^m/m^*ESC lines (Supplementary Figure S3B). The further shift to the right in the PC1 axis also suggests that *Tet1^−/−^* ESC lines have a more distinct global transcriptomic profile than *Tet1^+/+^* and *Tet1^m/m^* ESC lines. Consistently, we found that a large number of genes (1247) were significantly deregulated in *Tet1^−/−^* ESCs (vs. wild type) in contrast to only 744 genes in *Tet1^m/m^* ESCs (vs. wild type), with 169 genes in common (Figure 2B). We classified genes that were significantly deregulated in *Tet1^−/−^* ESCs but not in *Tet1^m/m^* ESCs (1078 genes) as genes influenced by the non-catalytic functions of Tet1. Most of these genes showed no or much smaller changes in *Tet1^m/m^* ESCs (vs. wild type), with 62% showing a statistically significant difference between *Tet1^−/−^* and *Tet1^m/m^* ESCs. Remarkably, of these 1078 genes, a vast majority (722 genes, 67%) were upregulated while a smaller fraction (356 genes, 33%) were downregulated (Figure 2C), suggesting that the non-catalytic functions of Tet1 primarily contribute to gene repression in ESCs. Gene Ontology (GO) analysis revealed that upregulated genes were mostly enriched in developmental processes including stem cell differentiation, lineage specification, placenta formation and signaling pathways such as TGF-β and MAPK, while downregulated genes were enriched in housekeeping processes such as metabolic processes (Figure 2D and Supplementary Figure S3C). Many of the genes aberrantly upregulated only in *Tet1^−/−^* ESCs were developmental genes. In ESCs, most developmental genes are bivalent, marked by activating H3K4me3 and suppressive H3K27me3 modifications rendering them poised for activation upon differentiation (56). We found that 1/3 of genes uniquely deregulated in *Tet1^−/−^* ESCs (364/1078) were bivalent genes (Figure 2E). These included aberrantly upregulated mesendoderm (*Gata2, Gata4, Gata6, Bin1*) and trophectoderm (*Cdx2, Hand1, Krt7, Eomes*) genes as well as downregulated ectodermal (*Pax6, Otx2*) genes (Figure 2F). The expression of many of these genes were confirmed by RT-qPCR as shown previously (Figure 1A). There was also a small overlap between the 744 DEGs in *Tet1^m/m^* ESCs and bivalent genes (119/744), but it was not statistically significant (Supplementary Figure S3D). This finding suggests that Tet1, regulates a fraction of bivalent developmental genes by preventing their untimely activation, largely in a catalytically-independent fashion.

### Wild type and catalytic mutant Tet1 occupy bivalent gene promoters

To identify which of the deregulated genes are directly bound and regulated by Tet1, we mapped the genomic occupancy of wild type and catalytic mutant Tet1 in ESCs by Cleavage Under Targets and Tagmentation (CUT&Tag). This technique, which is an alternative to ChIP, allows for robust identification of binding sites of chromatin-bound proteins or histone modifications with minimal background noise (41). We introduced a 3xFlag epitope tag before the stop codon of the *Tet1* gene in *Tet1^+/^^+^* and *Tet1^m/m^* ESCs and generated homozygous *Tet1^+/+^^;^^F^^lag/^^F^^lag^* and *Tet1^m/m;^^F^^lag/^^F^^lag^* ESCs to facilitate mapping Tet1 occupancy using an anti-Flag antibody (Figure 2G). To ensure antibody specificity, we used an untagged ESC line and IgG as negative controls. The CUT&Tag analysis identified a total of 26012 and 22203 peaks in *Tet1^+/+;^^F^^lag/^^F^^lag^* and *Tet1^m/m;^^F^^lag/^^F^^lag^* ESCs respectively, with 17283 overlapping (Figure 2G). To gain further confidence and reproducibility in our analysis, we also applied CUT&Tag to map Tet1 occupancy in *Tet1^+/+^ and Tet1^m/m^* ESCs using an anti-Tet1 antibody and *Tet1^−/−^* ESCs as negative control (Supplementary Figure S3E). We found a strong concordance between the two datasets (*r* = 0.9; 76% overlap of the called peaks) (Supplementary Figure S3F), suggesting that the CUT&Tag method and the Flag tag uncovered bona fide Tet1 genomic distribution. The distribution of Tet1 wild type and Tet1 mutant peaks were very similar across all genomic regions, with majority of the peaks (∼40%) located at promoters (± 2 kb of transcription start site (TSS) (Figure 2H and I). Tet1 wild type and mutant promoter peaks were mapped to 7549 genes of which 1671 were bivalent genes (1/3 of all bivalent genes in ESCs) (Figure 2J). We found that over half (562/1078) of genes that are uniquely deregulated in *Tet1^−/−^* but not in *Tet1^m/m^* ESCs, were bound by Tet1 at their promoters (i.e. direct non-catalytic targets of Tet1). Likewise, 204/364 deregulated bivalent genes were directly bound by Tet1 (i.e. direct non-catalytic bivalent targets of Tet1) (Figure 2J) including key lineage specifier genes such as *Cdx2, Foxa2 and Gata4* (Figure 2K and Supplementary Figure S3G). This analysis confirmed that both wild type and catalytic mutant Tet1 have similar genomic occupancy and are enriched at promoters of a fraction of bivalent genes to contribute to their repression.

### Tet1 and chromatin modifying complexes co-occupy bivalent and active gene promoters to regulate gene expression in a non-catalytic manner

The majority of Tet1 non-catalytic target genes (genes bound by Tet1 and deregulated in *Tet1^−/−^* but not in *Tet1^m/m^* ESCs) were upregulated genes. This prompted us to examine if Tet1 non-catalytic target genes are also targets of chromatin repressive complexes. Previous studies have reported co-occupancy of Tet1 and chromatin repressive complexes PRC2 and Sin3a (21,23). However, the extent to which Tet1 cooperates catalytically versus non-catalytically with these complexes is not well-established. We overlapped Tet1 non-catalytic target genes with genes bound by Ezh2 (PRC2 component and H3K27 trimethyl transferase), Sin3a (component of histone deacetylase complex) and Chd4 (ATPase helicase and component of NuRD chromatin remodeling complex) using published ESC ChIP-seq datasets described in methods. We found that a significant fraction of Tet1 non-catalytic target genes were directly bound by Ezh2 (130/562, ∼25%), Sin3a (191/562, 34%) and Chd4 (351/562, 62%) (Supplementary Figure S4A). This, along with some similarities between differentiation defects of *Tet1^−/−^* ESCs and ESCs deficient for these epigenetic regulators (57–60), prompted us to pursue the relationship between Tet1, Ezh2, Sin3a and Chd4 in detail and assess their impact on establishing bivalency and proper gene repression.

**Figure 3.**
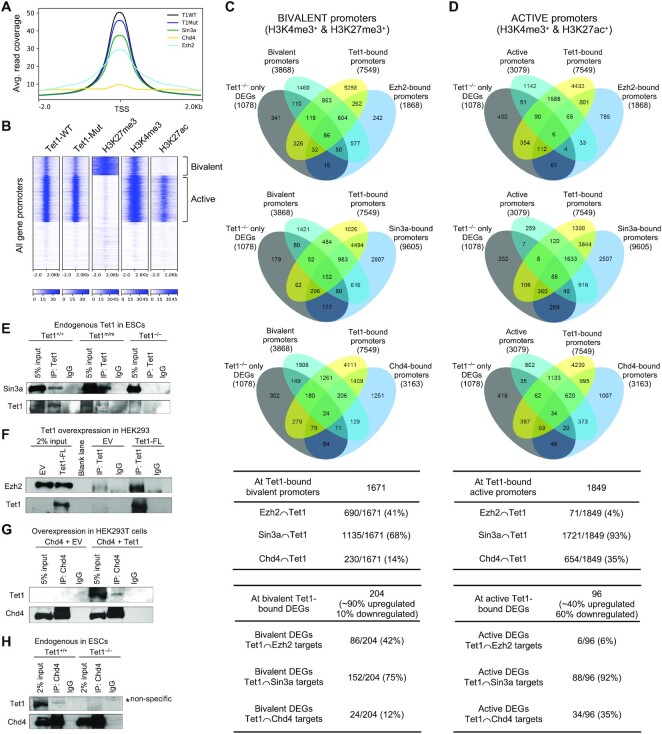
Enrichment of Tet1, Sin3a, Ezh2 and Chd4 at bivalent and active promoters. (**A**) Co-occupancy of Tet1 wild type (Tet1-WT) and Tet1 catalytic mutant (Tet1-Mut), Ezh2, Sin3a and Chd4 at all gene promoter regions (±2 kb of TSS). Each line represents the average read coverage over all gene promoter regions. 23922 gene promoters from the mm10 genome were analyzed. (**B**) Heatmaps illustrating enrichment of Tet1, H3K27me3, H3K4me3 and H3K27ac CUT&Tag signals at all gene promoters (±2 kb of TSS). Promoters were clustered by *K*-means method based on CUT&Tag read density using seqMINER software. (**C**) Overlap of bivalent, Tet1-bound, Ezh2-bound, Sin3a-bound, Chd4-bound promoters, and genes differentially expressed in *Tet1^−/−^* ESCs only, depicted in Venn diagram (top) and listed (bottom). (**D**) Overlap of active, Tet1-bound, Ezh2-bound, Sin3a-bound, Chd4-bound promoters, and genes differentially expressed in *Tet1^−/−^* ESCs only, depicted in Venn diagram (top) and listed (bottom). (**E**) Co-immunoprecipitation of endogenous Tet1 with Sin3a in *Tet1^+/+^* and *Tet1^m/m^* ESCs using anti-Tet1 antibody. *Tet1^−/−^* ESCs are used as negative control. (**F**) Co-immunoprecipitation of exogenously expressed full length Tet1 (Tet1-FL) with Ezh2 in HEK293T cells using anti-Tet1 antibody. Cells expressing empty vector (EV) are used as a negative control. (**G**) Co-immunoprecipitation of exogenously expressed full length Tet1 (Tet1-FL) with Chd4 in HEK293T cells using anti-Chd4 antibody. Cells expressing empty vector (EV) are used as a negative control. (**H**) Co-immunoprecipitation of endogenous Tet1 with Chd4 in wild type ESCs using anti-Chd4 antibody. *Tet1^−/−^* ESCs are used as negative control.

We mapped the genome-wide occupancy of Ezh2, bivalent marks (H3K27me3 and H3K4me3), Sin3a, H3K27ac and Chd4, in *Tet1^+/+^*, *Tet1^m/m^* and *Tet1^−/−^* ESCs by CUT&Tag or CUT&RUN (Supplementary Figure S4B). We found that Tet1 occupancy at TSS (±2 kb) strongly overlapped with Ezh2 and Sin3a and to a lesser degree with Chd4 (Figure 3A). Both wild type and catalytic mutant Tet1 primarily associated with bivalent promoters (marked with activating H3K4me3 and repressive H3K27me3 marks) and active promoters (marked with H3K4me3 and H3K27ac) (Figure 3B). About 43% of bivalent promoters (1671/3868) were bound by Tet1. 41% (690/1671) of Tet1-bound bivalent promoters were bound by Ezh2 and 68% (1135/1671) were bound by Sin3a suggesting a strong enrichment of Tet1, Ezh2 and Sin3a at bivalent promoters (Figure 3C). Importantly, 90% of the bivalent Tet1-bound DEGs were upregulated in *Tet1^−/−^* ESCs, and a remarkable 42% and 75% of them were bound by Ezh2 and Sin3a, respectively, strongly associating occupancy of these proteins with gene repression (Figure 3C). In contrast, Chd4-bound genes constituted a small fraction of Tet1-bound bivalent genes (14%) and Tet1-bound bivalent DEGs (12%) (Figure 3C). However, Tet1-bound active promoters exhibited 35% overlap with Chd4-bound gene promoters and 93% overlap with Sin3a-bound gene promoters in contrast to only 4% overlap with Ezh2-bound gene promoters. Consistently, of the 96 active Tet1-bound DEGs a large fraction were bound by Chd4 and Sin3a, but not by Ezh2 (Figure 3D).

Given the significant co-occupancy between Tet1, Ezh2, Sin3a and Chd4, we tested whether Tet1 is in a complex with any of these chromatin modifiers. We found that Sin3a co-immunoprecipitated with both wild type and catalytic mutant Tet1 in *Tet1^+/+^* and *Tet1^m/m^* ESCs, respectively. This expands on previous observations (21) by showing that the Tet1–Sin3a complex formation is not dependent on catalytic activity (Figure 3E). In contrast, Ezh2 did not co-immunoprecipitate with Tet1 in ESCs, but was only detected in immunoprecipitates of overexpressed Tet1 in HEK293T cells (Figure 3F). Likewise, Chd4 and Tet1 co-immunoprecipitated with low efficiency both in ESCs and when overexpressed in HEK293T cells (Figure 3G and H). In all co-immunoprecipitation assays ESC nuclear extracts were treated with the endonuclease benzonase before protein purification to rule out the possibility that any weak interaction between proteins is mediated by interceding DNA. Our findings support a robust and strong catalytic independent complex formation between Tet1 and Sin3a compared to Ezh2 and Chd4. This is consistent with the strong co-occupancy of Tet1 and Sin3a at bivalent and active promoters.

### Loss of Tet1 compromises Ezh2/Sin3a enrichment and H3K27me3/H3K27ac deposition at bivalent genes in a non-catalytic fashion

**Figure 4.**
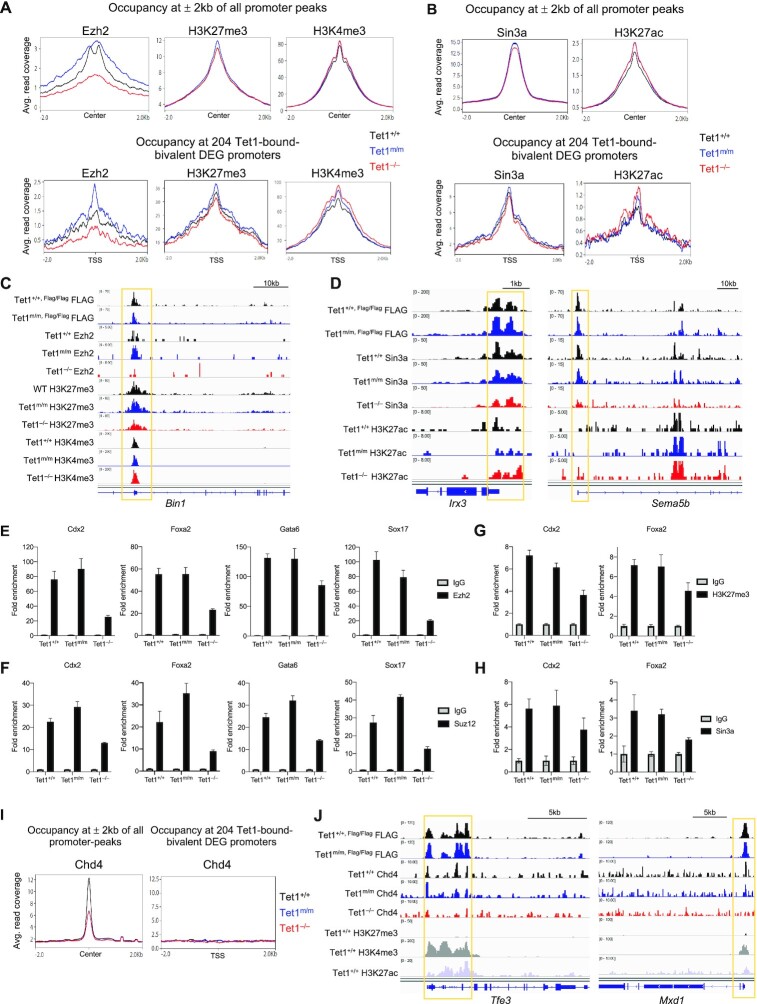
Comparison of enrichment of Ezh2, Sin3a, Chd4 and histone modifications at bivalent gene promoters in *Tet1^+/+^*, *Tet1^m/m^* and *Tet1^−/−^* ESCs. (**A**) Enrichment of Ezh2, H3K27me3 and H3K4me3 CUT&Tag signals at all promoter peaks (± 2 kb from the center of peaks) (top), and at 204 Tet1-bound bivalent differentially expressed gene promoters (±2 kb of TSS) (bottom) in *Tet1^+/+^*, *Tet1^m/m^* and *Tet1^−/−^* ESCs. (**B**) Enrichment of Sin3a and H3K27ac CUT&Tag signals at all promoter peaks (± 2 kb from the center of peaks) (top), and at 204 Tet1-bound bivalent differentially expressed gene promoters (± 2 kb of TSS) (bottom) in *Tet1^+/+^*, *Tet1^m/m^* and *Tet1^−/−^* ESCs. (**C**) Genome browser tracks showing enrichment of Tet1, Ezh2, H3K27me3 and H3K4me3 at regulatory regions of a selected Tet1 target gene in *Tet1^+/+^*, *Tet1^m/m^* and *Tet1^−/−^* ESCs. (**D**) Genome browser tracks showing enrichment of Tet1, Sin3a and H3K27ac at regulatory regions of selected Tet1 target genes in *Tet1^+/+^*, *Tet1^m/m^* and *Tet1^−/−^* ESCs. (**E-H**) Quantification of enrichment of Ezh2, Suz12, H3K27me3 and Sin3a at regulatory elements of indicated developmental genes by ChIP-qPCR in *Tet1^+/+^*, *Tet1^m/m^* and *Tet1^−/−^* ESCs (data normalized to 10% input control and IgG). Three independent ESC lines of each genotype were used. (**I**) Enrichment of Chd4 CUT&RUN signals at all promoter peaks (±2 kb from the center of peaks) (left), and at 204 Tet1-bound bivalent differentially expressed gene promoters (± 2 kb of TSS) (right) in *Tet1^+/+^*, *Tet1^m/m^* and *Tet1^−/−^* ESCs. (**J**) Genome browser track snapshots showing enrichment of Tet1, Chd4 and promoter histone marks at regulatory regions of selected active Tet1 target genes in *Tet1^+/+^*, *Tet1^m/m^* and *Tet1^−/−^* ESCs.

To establish how loss of Tet1 vs. loss of its catalytic activity leads to aberrant gene activation in ESCs, we compared the enrichment of Ezh2 and Sin3a and the deposition of H3K27me3 and H3K27ac in *Tet1^−/−^* versus *Tet1^m/m^* and *Tet1^+/+^* ESCs. We found reduced Ezh2 enrichment at all gene promoters, specifically at Tet1-bound bivalent DEG promoters, in *Tet1^−/−^* ESCs but not *Tet1^m/m^* or *Tet1^+/+^* ESCs (Figure 4A). While H3K27me3 enrichment at all promoters was not severely affected, it was diminished at Tet1-bound bivalent DEGs in *Tet1^−/−^* ESCs consistent with reduced Ezh2 enrichment. H3K4me3 levels were largely unchanged at all promoters, but there was a subtle increase at Tet1-bound bivalent DEGs in *Tet1^−/−^* ESCs versus *Tet1^m/m^* and *Tet1^+/+^* ESCs (Figure 4A). This suggests that Tet1 facilitates recruitment of Ezh2 in a catalytic independent fashion for H3K27me3 deposition and gene repression. Indeed treating wild type ESCs with the Ezh2 inhibitor GSK-126 upregulated bivalent genes such as *Eomes* (Supplementary Figure S5A) while treatment with H3K27 demethylase inhibitor GSK-J4 reversed aberrant bivalent gene upregulation in *Tet1^−/−^* ESCs (Supplementary Figure S5B). Taken together, these data confirm that aberrant bivalent gene upregulation in Tet1^−/−^ ESCs is due to reduced Ezh2 recruitment and H3K27 trimethylation. We also observed a subtle reduction in Sin3a recruitment at all promoters, including at Tet1-bound bivalent DEG promoters, in *Tet1^−/−^* ESCs compared to *Tet1^m/m^* and *Tet1^+/+^* ESCs. This was coupled with a subtle increase in H3K27ac levels at Tet1-bound bivalent DEGs in *Tet1^−/−^* ESCs, which could contribute to the aberrant upregulation of bivalent genes (Figure 4B). The compromised enrichment of Ezh2 and Sin3a and reduced H3K27 trimethylation and deacetylation in *Tet1^−/−^* ESCs, but not in *Tet1^m/m^* and *Tet1^+/+^* ESCs, was visualized at representative genome browser tracks of selected bivalent genes (Figure 4C and D) and supported by ChIP-qPCR at promoters of selected bivalent Tet1-bound developmental genes that are uniquely downregulated in *Tet1^−/−^* ESCs (Figure 4E–H). We found a profound reduction in enrichment of PRC2 components Ezh2 and Suz12 in *Tet1^−/−^* ESCs, but not in *Tet1^m/m^* and *Tet1^+/+^* ESCs, at promoters of *Cdx2, Foxa2, Gata6* and *Sox17* concomitant with reduced H3K27me3 levels (Figure 4E–G). Likewise, Sin3a enrichment at these genes was only reduced in *Tet1^−/−^* ESCs (Figure 4H). These findings suggest that Tet1, independent of its catalytic activity, recruits PRC2 and Sin3a to bivalent promoters, especially of the key developmental genes, to mediate gene repression via H3K27 deacetylation and trimethylation.

To examine whether Tet1 influences Chd4 recruitment to chromatin, we compared Chd4 occupancy in *Tet1^+/+^*, *Tet1^m/m^* and *Tet1^−/−^* ESCs by CUT&RUN. We found that Chd4 enrichment was reduced across all promoters in both *Tet1^−/−^* and *Tet1^m/m^* ESCs (Figure 4I). This implies that Chd4 recruitment to DNA could be influenced by DNA hydroxylation as is the case for recruitment of MBD3, another member of NuRD complex (61). Notably Chd4 occupancy at promoters of Tet1-bound bivalent DEGs was unchanged (Figure 4I and Supplementary Figure S5C) in agreement with poor overlap between Chd4-bound genes and Tet1-bound bivalent DEGs shown earlier (Figure 3C). However, consistent with the association of Chd4 with deregulated active genes, we found a diminished recruitment of Chd4 at promoters of active genes, like the pluripotency transcription factor *Tfe3* and *Mxd1*, in *Tet1^−/−^* ESC only (Figure 4J). Consistently, both of these genes were significantly downregulated only in *Tet1^−/−^* ESCs per RNA-seq data. Chd4 is a chromatin helicase and a member of the NuRD remodeling complex that can influence chromatin accessibility (58). We therefore compared chromatin accessibility in *Tet1^+/+^*, *Tet1^m/m^* and *Tet1^−/−^* ESCs by ATAC-seq and found only 28 differentially accessible regions (DARs) in *Tet1^m/m^* and 658 DARs in *Tet1^−/−^* compared to control wild type ESCs (Supplementary Figure S5D). Although this represents very few DARs, nearly half of all DARs (165/306-gained, 146/306-lost accessibility) fall in regions bound by Tet1, implying that Tet1 may impact accessibility to a certain degree. However, it must be noted that these minor changes in accessibility did not have a significant effect on gene expression and did not involve bivalent genes (Supplementary Figure S5E and F).

### Loss of Tet1 catalytic activity leads to global DNA hypermethylation at promoters and canyons and correlates with gene silencing but not with deregulation of bivalent genes

**Figure 5.**
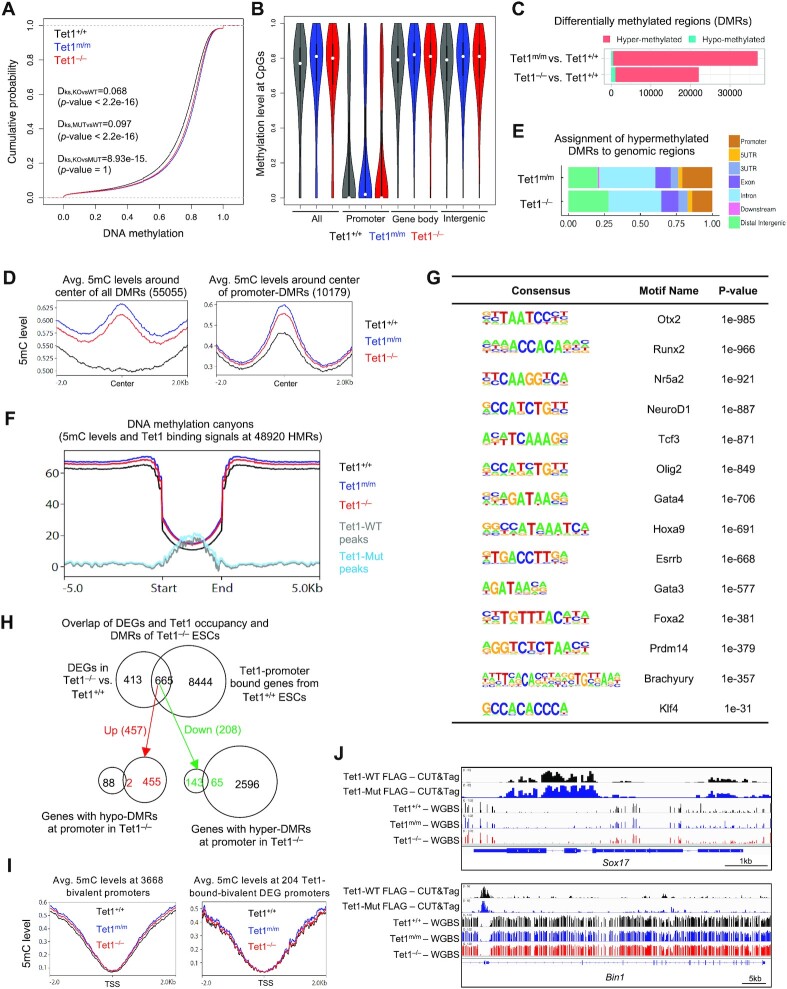
Genome-wide analysis of DNA methylation in *Tet1^+/+^*, *Tet1^m/m^* and *Tet1^−/−^* ESCs reveals no changes in methylation levels at bivalent genes. (**A**) Cumulative plot of average DNA methylation value within 100 bp-tiles across the mouse genome in *Tet1^+/+^*, *Tet1^m/m^* and *Tet1^−/−^* ESCs. Two independent lines of each genotype were used. Kolmogorov-Smirnov test was used to test if methylation levels were significantly different than *Tet1^+/+^* ESCs. (**B**) CpG methylation level over all genome and at different genomic regions including promoters, gene bodies and intergenic regions. (**C**) DMRs (differentially methylated regions) from *Tet1^m/m^* vs. *Tet1^+/+^* and *Tet1^−/−^* vs. *Tet1^+/+^* by thresholds of consecutive CpG sites >3 with an average methylation difference ≥10% for each region. (**D**) Average DNA methylation levels over ±2 kb of all DMRs (left) and promoter-DMRs (right) in *Tet1^+/+^*, *Tet1^m/m^* and *Tet1^−/−^* ESCs. (**E**) Assignment of hypermethylated DMRs to genomic regions. (**F**) 5mC levels and Tet1 binding at DNA methylation canyons in ESC lines of indicated genotypes. All 48920 hypo methylated regions (HMRs) in wild type ESCs were used to call canyons for this analysis. (**G**) Enrichment of developmental and pluripotency factor motifs in 12631 common DMRs from *Tet1^−/−^* vs. *Tet1^+/+^* and *Tet1^m/m^* vs. *Tet1^+/+^*. (**H**) Venn diagrams depicting overlap of genes uniquely deregulated in *Tet1^−/−^* ESCs and bound by Tet1 at their promoters with genes containing hyper- or hypo-DMRs at their promoters. (**I**) Average methylation levels at promoters (over ±2 kb of TSS) of all bivalent genes (left) and 204 Tet1-bound-bivalent differentially expressed genes (right). (**J**) Representative genome browser tracks showing wild type and catalytic mutant Tet1 occupancy and 5mC levels at selected bivalent genes in *Tet1^+/+^*, *Tet1^m/m^* and *Tet1^−/−^* ESCs. Note no change in methylation at Tet1-bound bivalent genes upon loss of Tet1 or loss of its catalytic activity.

Since Tet1 catalytic activity promotes DNA demethylation, we assessed the DNA methylation landscape of *Tet1^m/m^* and *Tet1^−/−^* ESCs in comparison to wild type ESCs by whole genome bisulfite sequencing (WGBS) (Supplementary Figure S6A). We found that global DNA methylation was similarly increased in both *Tet1^−/−^* and *Tet1^m/m^* ESCs compared to wild type ESCs (Figure 5A) with percent-methylated CpGs elevated across promoters, gene bodies and intergenic regions (Figure 5B). We identified differentially methylated regions (DMRs) in *Tet1^−/−^* and *Tet1^m/m^* ESCs (compared to wild type ESCs). DMRs were defined as regions containing at least 3 methylated CpGs within a maximum distance of 300bp with a methylation difference of at least 10%. We found 22094 DMRs in *Tet1^−/−^* ESCs (21042 hyper and 1052 hypo) and 36968 DMRs in *Tet1^m/m^* ESCs (36484 hyper and 484 hypo) with 12742 common DMRs (12631 hyper and 111 hypo) (Figure 5C and Supplementary Figure S6B). Presence of more DMRs in *Tet1^m/m^* than in *Tet1^−/−^* ESCs could be because binding of Tet1 mutant protein at these sites prevents any compensatory Tet2-mediated demethylation. A vast majority of DMRs were hypermethylated in both cell types consistent with a role for Tet1 catalytic activity in DNA demethylation. The methylation difference was pronounced at the center and along the ±2 kb of all DMRs (Figure 5D), with majority of the DMRs being ∼10% hypermethylated (Supplementary Figure S6C) suggesting that loss of Tet1 does not lead to robust DNA hypermethylation consistent with the fact that Tet1 only contributes to 30% of 5hmC in ESCs. The hypermethylated DMRs in both *Tet1^−/−^* and *Tet1^m/m^* ESCs largely mapped to introns and distal intergenic regions followed by promoters and exons (Figure 5E) and were also enriched at DNA methylation canyons and repetitive elements (Figure 5F and Supplementary Figure S6D). Canyons are large hypomethylated regions in euchromatin that harbor many pluripotency and differentiation genes. Tet1 is enriched at the canyons presumably to maintain a hypomethylated state and proper gene expression (62,63). We found that 5mC levels were equally elevated at canyons in both *Tet1^−/−^* and *Tet1^m/m^* ESCs suggesting that Tet1 catalytic activity regulates DNA methylation at canyons. We also found that DMRs in *Tet1^−/−^* and *Tet1^m/m^* ESCs were enriched for binding motifs of many stemness and differentiation master transcription factors (Figure 5G). This suggests that Tet1 catalytic activity keeps the DNA binding sites of master transcription factors in a hypomethylated state in ESCs.

Given that Tet1 is enriched at promoters and a significant fraction of hypermethylated DMRs in *Tet1^m/m^* and *Tet1^−/−^* ESCs mapped to promoters, we examined how this influences gene expression. To this end, we compared Tet1 promoter-bound down- or upregulated genes with genes containing hyper- or hypomethylated DMRs at their promoters. While ∼1/3 of downregulated genes had hypermethylated DMRs in their promoters, almost none of the upregulated genes contained hypomethylated DMRs (Figure 5H) suggesting that promoter DNA hypermethylation contributes to gene silencing while DNA hypomethylation does not correlate with the aberrant upregulation of Tet1 target genes. Since bivalent genes are uniquely upregulated in *Tet1^−/−^*, but not in *Tet1^m/m^* ESCs, we specifically examined DNA methylation changes at bivalent genes in *Tet1^m/m^* and *Tet1^−/−^* ESCs. We found no significant difference in DNA methylation levels at promoters of all bivalent genes and the 204 Tet1-target bivalent genes that were uniquely deregulated in *Tet1^−/−^* ESCs (Figure 5I and J). Likewise, we did not find any notable overlap between deregulated bivalent genes and genes with hyper- or hypomethylated promoters (Supplementary Figure S6E). This suggests that aberrant activation of bivalent genes is not due to any impaired DNA demethylation or loss of Tet1 catalytic activity, and instead is mainly regulated by Tet1 non-catalytic functions.

### Tet1 knockout, but not catalytic mutant, blastocysts and mid-gestation embryos are smaller in size and developmentally delayed

**Figure 6.**
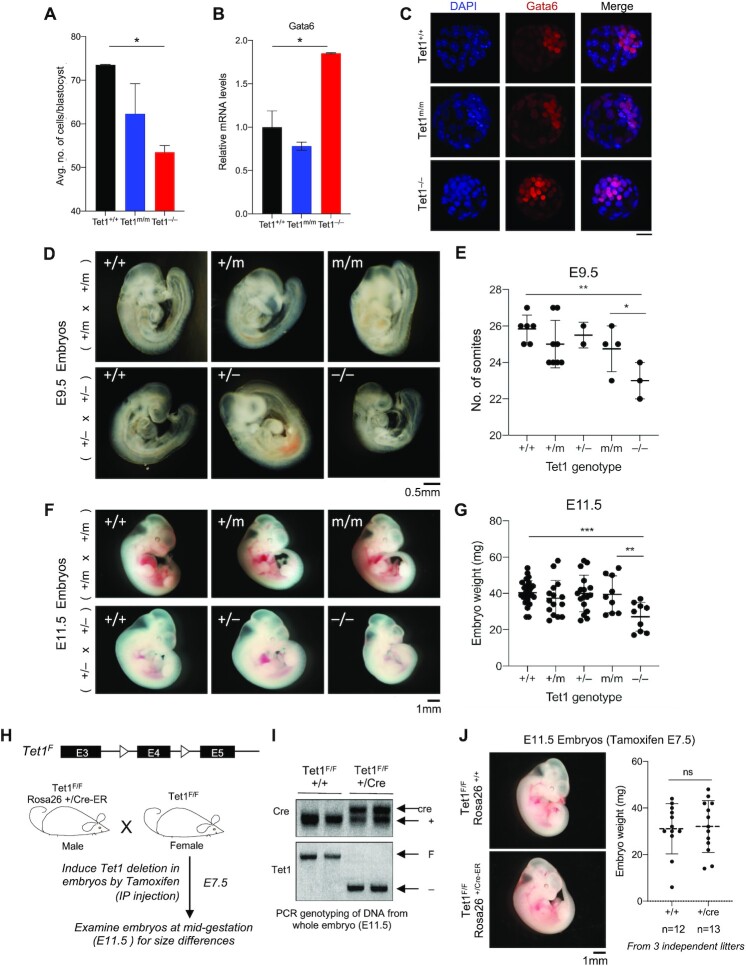
Comparative analysis of *Tet1^+/+^*, *Tet1^m/m^* and *Tet1^−/−^* blastocysts and mid-gestation embryos. (**A**) Unbiased quantification of blastocyst cell numbers by Volocity 3D Image Analysis software. *n* = 4 blastocysts per genotype were analyzed. Statistically significant * *P* < 0.05 (**B**) Quantification of *Gata6* mRNA levels in blastocysts by RT-qPCR. *n* = 4 blastocysts of each genotype were analyzed. Data normalized to *Gapdh*. Error bars represent SEM. One way ANOVA test was used to assess statistically significant differences * *P* < 0.05. (**C**) Immunostaining for Gata6 (red) and nuclei (blue) in *Tet1^+/+^*, *Tet1^m/m^* and *Tet1^−/−^* blastocysts. Scale bar = 27 μm. (**D**) Gross images of E9.5 embryos of the indicated Tet1 genotypes. Scale bar = 0.5 mm (**E**) Somite counts of E9.5 embryos of the indicated genotypes. Each dot represents an embryo. Error bars represent SD. One way ANOVA test was used to assess statistically significant differences * *P* < 0.05, ** *P* < 0.01. (**F**) Gross images of E11.5 embryos of the indicated Tet1 genotypes. Scale bar = 1 mm (**G**) Weight of E11.5 embryos of the indicated genotypes. Each dot represents an embryo. Error bars represent SD. One way ANOVA test was used to assess statistically significant differences, ** *P* < 0.01, *** *P* < 0.001 (**H**) Schematic of breeding strategy for inducible deletion of Tet1 during embryogenesis. (**I**) PCR genotyping of embryos confirming complete excision of *Tet1* exon 4 in Cre-positive E11.5 embryos (tamoxifen treated E7.5). (**J**) Representative gross images of E11.5 *Tet1^F/F^Rosa26^+/+^* and *Tet1^F/F^Rosa26^+/Cre-ER^* embryos (left). Weight of E11.5 embryos of indicated genotypes. Each dot represents an embryo. Three independent litters were analyzed. Error bars represent SD. Unpaired *t*-test was used to assess statistically significant differences, ns = not significant.

Tet1 is highly expressed during early embryonic development, in particular in the inner cell mass of the blastocyst from where ESCs are derived. Previously, we have shown that loss of Tet1 in mice in a mixed 129/B6 background delays embryonic development leading to smaller-sized mid-gestation embryos, neonates and adults (12). To examine how the catalytic versus non-catalytic roles of Tet1 influence embryonic development, we generated *Tet1^+/−^* and *Tet1^+/m^* mice in mixed 129/B6 background and intercrossed each strain to obtain and analyze *Tet1^−/−^* and *Tet1^m/m^* pre- and post-implantation embryos. We found that *Tet1^−/−^*, but not *Tet1^m/m^*, blastocysts were smaller in size with fewer cells and, consistent with *Tet1^−/−^* ESCs expressed higher levels of the bivalent gene and mesendoderm marker Gata6 (Figure 6A–C). Both *Tet1^−/−^* and *Tet1^m/m^* mid-gestation embryos at E9.5 and E11.5 developed with expected mendelian ratios (Supplementary Figure S7A). However, *Tet1^−/−^*, but not *Tet1^m/m^*^,^ embryos had significantly fewer somites, weighed less, and were smaller in size (Figure 6D–G). This expands on our prior work (12) by demonstrating that the non-catalytic functions of Tet1 are essential for preventing developmental delay.

Since Tet1 is highly expressed in the blastocyst to epiblast stages of embryogenesis, we wanted to establish whether the effects of Tet1 loss on embryonic development is due to loss of Tet1 during these early stages or later in embryogenesis. To this end we deleted Tet1 in *Tet1^f/f^ Rosa26^+/CreER^* embryos at E7.5 by tamoxifen administration and analyzed them at E11.5 (Figure 6H and I). We found that *Tet1^f/f^ Rosa26^+/CreER^* and littermate control *Tet1^f/f^ Rosa26^+/+^* embryos were indistinguishable in size and weight (Figure 6J). This suggests that loss of Tet1 in post-gastrulation embryos does not lead to developmental delay, and that the reduced growth observed in germline *Tet1^−/−^* mice is likely caused by Tet1 deficiency early in development, consistent with higher expression of Tet1 during the blastocyst stage.

To assess the catalytic and non-catalytic requirements of Tet1 during late gestation and postnatal development, we intercrossed *Tet1^+/−^* or *Tet1^+/m^* mice to generate and compare *Tet1^−/−^* and *Tet1^m/m^* E16.5 embryos as well as postnatal adults. Both *Tet1^−/−^* and *Tet1^m/m^* E16.5 embryos were viable and developed at expected mendelian ratios, and their weights and sizes were comparable but significantly smaller than littermate heterozygote and wild type embryos (Supplementary Figure S7B-D). Postnatal *Tet1^−/−^* mice and *Tet1^m/m^* mice survived to weaning below the expected mendelian ratio (*Tet1^−/−^* = 17.6%, *Tet1^m/m^*= 16.8% versus expected 25%) (Supplementary Figure S7E), suggesting that 1/3 of both *Tet1^−/−^* and *Tet1^m/m^* die perinatally or as neonates before weaning. The surviving adult mice of both genotypes were comparable in size but slightly smaller than littermate heterozygote and wild type mice (Supplementary Figure S7F). These findings suggest that the catalytic activity of Tet1 is likely more important in late gestation and postnatal development, while the non-catalytic functions are more critical in early embryonic development consistent with a non-catalytic role in regulation of early developmental and lineage-specific genes.

## DISCUSSION

**Figure 7.**
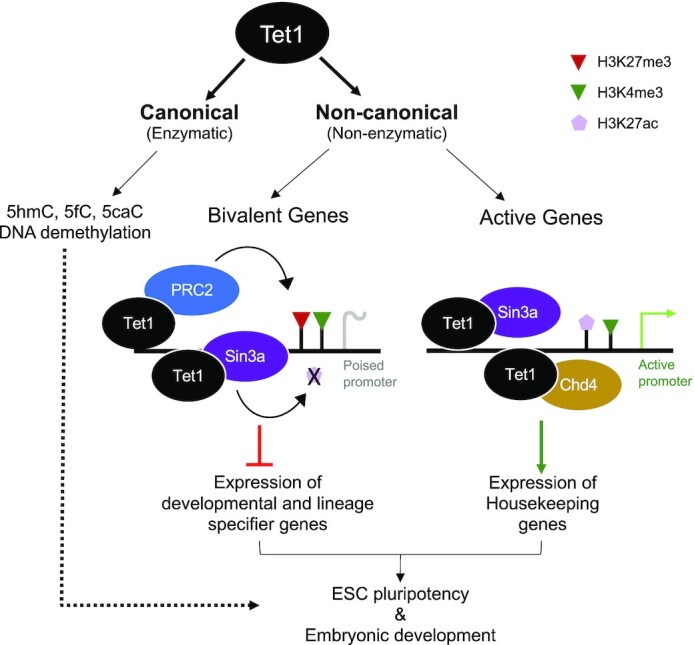
Model for the dual catalytic dependent and independent requirements of Tet1 in embryonic stem cell biology and development. Tet1 independent of its catalytic activity suppresses developmental genes by facilitating Sin3a-mediated H3K27 deacetylation and PRC2-mediated H3K27 trimethylation at gene promoters.

Since its discovery as a DNA dioxygenase many studies have investigated the biological functions of Tet1 in ESCs and development. However, dissecting the contributions of its canonical functions (i.e. DNA demethylation) vs. non-canonical roles (i.e. formation of chromatin regulatory complexes) in regulation of ESCs and embryogenesis has been a long standing goal in the field. This is, in part, due to utilization of knockdown and knockout approaches which deplete the entire protein and fail to distinguish its enzymatic and non-enzymatic roles. We have circumvented this problem by generating Tet1 catalytic deficient ESCs and mice using defined mutations in the iron binding pocket of Tet1 which renders it catalytically inactive. These mutations fully abrogate the enzymatic activity of Tet1 and do not act in a dominant negative fashion as heterozygote ESCs and mice are devoid of any phenotypes and are comparable to their wild type counterparts. Our comparative analysis of Tet1 catalytic mutant and knockout ESCs and mice provides four lines of evidence which supports prominent catalytic independent gene regulatory roles and biological requirements for Tet1 in ESCs and during early development. We have shown that loss of Tet1, but not loss of its catalytic activity leads to (i) aberrant activation of developmental regulators including bivalent genes, (ii) differentiation defects along mesendoderm and trophectoderm lineages, (iii) early embryonic developmental delay, (iv) impaired enrichment of PRC2 and Sin3a at bivalent gene promoters leading to reduced H3K27 trimethylation and H3K27 deacetylation. This work establishes critical requirements for non-canonical functions of Tet1 in ESC gene regulation, differentiation and development (Figure 7). It shifts the paradigm on how Tet1 regulates ESCs by highlighting its prominent biological functions that go beyond DNA demethylation.

We identified 1078 genes in ESCs that are regulated by Tet1 non-catalytic functions. The expression changes of these genes are only significant in *Tet1^−/−^* ESCs (vs. *Tet1^+/+^*), as assessed by PCA and other statistical analyses. Furthermore, the extent of changes in *Tet1^m/m^* ESCs is much smaller or negligible. Hence, the transcriptomic differences of *Tet1^−/−^* and *Tet1^m/m^* ESCs are not artifacts of statistical thresholds. It is not caused by expansion of differentiated cells in *Tet1^−/−^* colonies either, as *Tet1^−/−^* ESCs exhibit comparable expression of pluripotency markers to *Tet1^+/+^* and *Tet1^m/m^* ESCs. Interestingly, the majority of the 1078 genes regulated by non-catalytic functions of Tet1 are repressed genes directly bound by Tet1 at their promoters. While loss of Tet1 has been implicated in gene repression involving Sin3a and PRC2 previously (21,23), our work conclusively shows that Tet1-mediated gene repression mechanisms are entirely catalytic-independent. Tet1 non-catalytic target genes include developmental regulators of the mesendoderm and trophectoderm lineages that are bivalently marked (H3K4me3^+^ and H3K27me3^+^) and are poised for activation. Our findings that wild type and catalytic mutant Tet1 have similar genomic occupancy, including at bivalent and active promoters, suggests that catalytic activity is not essential for targeting Tet1 to the chromatin. In agreement with previous Tet1 ChIP-seq studies (21,23), we find that Tet1 shows co-occupancy with distinct epigenetic modifiers depending on promoter type. Wild type or catalytic mutant Tet1 co-occupies bivalent promoters along with the PRC2 component Ezh2 and the Sin3a deacetylase complex. Consistently, we find wild type or catalytic mutant Tet1 to be in a complex with Sin3a and may transiently interact with Ezh2. This shows that catalytic activity is not essential for formation of these complexes, expanding on prior studies that have reported these interactions (21,64). While loss of Tet1 did not affect genome-wide targeting of Sin3a and Ezh2, it diminished their enrichment specifically at bivalent promoters leading to reduced H3K27 deacetylation and trimethylation, respectively. In contrast to the robust Ezh2 reduction at gene promoters in *Tet1^−/−^* ESCs, the overall reduction in H3K27me3 levels was modest, suggesting that low levels of Ezh2 can still promote sufficient H3K27 trimethylation. However, at specific bivalent developmental genes that we have identified (e.g. *Foxa2* and *Cdx2*), H3K27me3 levels were substantially reduced as captured by ChIP-qPCR and supported by the enrichment signals at those loci as shown in genome browser tracks. Since neither Sin3a/Ezh2 enrichment nor H3K27 deacetylation/trimethylation was affected at bivalent genes in Tet1 catalytic-deficient ESCs, it convincingly establishes that Tet1-mediated facilitation of Sin3a and PRC2 recruitment and subsequent H3K27 modifications are entirely catalytic-independent processes. Moreover, H3K27 trimethylation is sufficient for repression of bivalent genes as we find that chemical modulation of H3K27me3 levels alone is enough to induce aberrant activation of selected bivalent genes, mimicking Tet1 loss. We also note that some molecular and phenotypic signatures of *Tet1^−/−^*, but not *Tet1^m/m^*, ESCs resemble those of Ezh2- and Sin3a-deficient ESCs (57,59,60), further linking Tet1 to Ezh2- and Sin3a-mediated gene regulation. Interestingly, we note wider Ezh2 peaks in *Tet1^m/m^* ESCs than in *Tet1^+/+^* ESCs, which we speculate could be due the increased DNA methylation in *Tet1^m/m^* ESCs making Ezh2 binding more diffuse, and this is reflected as broadened peaks. This is not observed at bivalent gene promoters which are often in a hypomethylated state and not affected between the two genotypes. Future studies will be needed to further investigate this.

While Tet1-Ezh2 co-occupancy is seen at bivalent genes only, Tet1-Sin3a co-occupancy is seen at both bivalent and active genes. This is consistent with many Hdac1/2-containing complexes, like Sin3a, playing both transcriptional activation and repression roles (65). Tet1 and Sin3a co-occupy the majority of active promoters (H3K4me3^+^, H3K27ac^+^) which includes promoters of Tet1 non-catalytic active target genes that are uniquely downregulated in *Tet1^−/−^* ESCs. We also identify a novel Tet1-Chd4 co-occupancy at active promoters. Chd4 is an ATP-dependent chromatin remodeler and component of NuRD complex that is highly expressed in ESCs (58). Tet1 immunoprecipitated with Chd4, albeit weakly, and loss of Tet1, but not loss of its catalytic activity, diminished enrichment of Chd4 at active promoters and correlated with gene silencing. This implies that Tet1-Chd4 and Tet1-Sin3a associations at active genes can contribute to their proper expression in a catalytic independent manner.

Deficiency of Tet1 or of its catalytic activity alone, as expected, had subtle and comparable global effects on DNA methylation levels in ESCs with vast majority of the DMRs being 10–20% hypermethylated. Interestingly, we find more DMRs in *Tet1^m/m^* ESCs than in *Tet1^−/−^* ESCs. This could be due to binding of catalytic mutant Tet1 at certain regions preventing any compensatory effects by Tet2, which is also expressed in ESCs. Future work aimed at mapping Tet2 occupancy in *Tet1^−/−^* ESCs versus *Tet1^m/m^* ESCs may elaborate more on any compensatory demethylation by Tet2. Tet1 can bind unmethylated CpG rich regions and simply protect them from being methylated to maintain a hypomethylated state (21,27). This safeguard mechanism is presumably catalytic independent and while its impact is not seen at the global level in our data, it may influence specific genes and loci. Tet1 is also implicated in maintaining DNA methylation valleys or canyons in a hypomethylated state in ESCs to allow for proper expression of genes they harbor (62,63,66). Our findings that 5mC levels are comparably elevated at canyons in *Tet1^m/m^* and *Tet1^−/−^* ESCs suggests that canyon DNA methylation levels are regulated by Tet1 catalytic activity.

While there was a correlation with promoter hypermethylation and aberrant gene silencing, there was no association between DNA hypomethylation and aberrant gene activation. Specifically, DNA methylation levels at bivalent promoters were unaffected in both catalytic deficient and knockout ESCs, and the aberrantly activated genes in *Tet1^−/−^* ESCs did not have differential promoter methylation. This suggests that Tet1-mediated modulation of DNA methylation is not directly responsible for regulation of bivalent genes. This is consistent with literature showing that bivalent promoters are in hypomethylated regions in ESCs and since many are poised for rapid transcription upon stimulation they are not stably silenced by DNA methylation (23,67). This further supports that Tet1 regulates bivalent genes independently of its enzymatic activity. We also find that many of the DMRs are enriched for binding motifs of prominent developmental regulators and transcription factors. This suggests that while Tet1 may not directly regulate expression of developmental genes via methylation, it may influence their targeting to the chromatin by regulating methylation levels near their binding sites, as is shown for some transcription factors like Sall4 (68).

Our work shows that the non-catalytic functions of Tet1 have implications in ESC biology and embryogenesis. The non-catalytic target genes of Tet1 include developmental regulators of the mesendoderm and trophectoderm lineages. Deregulation of these classes of genes and abnormal differentiation towards extraembryonic lineages have been previously reported in *Tet1^−/−^* ESCs by us and others (12,15). Our findings that *Tet1^−/−^*, but not *Tet1^m/m^*, ESCs have skewed differentiation towards the trophectoderm lineage categorically establishes a non-catalytic requirement for Tet1 in safeguarding ESC identity and preventing aberrant ‘trans’ differentiation towards the extraembryonic lineage. This defines a critical non-catalytic role for Tet1 in regulating the epigenetic barriers between embryonic and extraembryonic fates with potential implications in cellular reprogramming and cell fate transitions. The biological significance of non-catalytic functions of Tet1 extends beyond ESCs into embryogenesis. Previously, we have shown that *Tet1^−/−^* mice are viable but smaller in size in a mixed 129/B6 background (12) and others have shown it to be lethal or sublethal in other genetic backgrounds (16,17). Our findings that *Tet1^−/−^* blastocysts in contrast to *Tet1^m/m^* blastocysts have increased expression of mesendoderm marker Gata6 is consistent with distinct upregulation of *Gata6* in *Tet1^−/−^* ESCs. This, together with the observation that *Tet1^−/−^* mid-gestation embryos are developmentally delayed, while their *Tet1^m/m^* counterparts are not, indicate an *in vivo* requirement for Tet1 non-catalytic functions in proper regulation of developmental genes and early embryonic development. Since inducible deletion of Tet1 at E7.5 does not affect embryo growth or size by mid-gestation, it is plausible to conclude that the absence of Tet1 non-catalytic functions early in development are likely responsible for the mild developmental delay of *Tet1^−/−^* embryos. Interestingly, late gestation and postnatal *Tet1^−/−^* and *Tet1^m/m^* mice are produced at similar mendelian ratios and are comparable in size (both smaller than wild type littermates). This suggests that while Tet1 catalytic activity is not critical in early embryogenesis, it has biological implications in late development and postnatally. Together, our data establishes a prominent requirement for Tet1 in early embryogenesis which is largely catalytic independent.

We conclude that the non-catalytic functions of Tet1 are essential for proper regulation of ESC gene expression programs, stem cell identity and plasticity, and embryonic development. Although this study has focused on ESCs with an emphasis on gene repression involving establishment of bivalency, it has implications for other stem cell types where Tet1 is expressed such as germ cells, neural stem cells and hematopoietic stem cells. Previously, we have shown that Tet2 has distinct catalytic dependent and independent functions in myeloid and lymphoid lineages, respectively (19), and it is likely that Tet1 has similar dual contributions in hematopoiesis. Our Tet1 catalytic mutant and knockout mice provide a viable platform to investigate regulatory roles of Tet1 in other stem cell types and biological processes as well as in the context of its other partners such as O-linked GlcNAc Transferase (OGT) (69). Identification of distinct biological functions of epigenetic regulators such as Tet enzymes is essential not only for understanding gene regulatory mechanisms during development but also for properly targeting them to enhance stem cell applications or designing therapeutics for diseases where Tet enzymes are mutated or dysregulated.

## DATA AVAILABILITY

The RNA-seq, CUT&Tag, CUT&RUN, WGBS and ATAC-seq data sets have been deposited in the Gene Expression Omnibus (GEO) database (Accession number GSE176389).

## Supplementary Material

gkac089_Supplemental_Files
