# Supplementary material for: The DNA dioxygenase Tet1 regulates H3K27 modification and embryonic stem cell biology independent of its catalytic activity
Source: Nucleic Acids Res. 2022 Feb 12;50(6):3169–89. doi: 10.1093/nar/gkac089 (PMC8989540; doi:10.1093/nar/gkac089)
Supplement: gkac089_Supplemental_Files [file gkac089_supplemental_files.zip › 4._Chrysanthou_et_al_Supplemental_Files_Final-.pdf]

SUPPLEMENTAL MATERIAL

The DNA dioxygenase Tet1 regulates H3K27 modification and embryonic stem cell biology independent of its catalytic activity

List of supplemental items:

Figure S1: Generation of Tet1 catalytic-mutant (*Tet1$^{m/m}$*) and Tet1-knockout (*Tet1$^{–/–}$*) mouse embryonic stem cells.

Figure S2: Characterization of Tet1 catalytic-mutant (*Tet1$^{m/m}$*) and Tet1-knockout (*Tet1$^{–/–}$*) mouse embryonic stem cells.

Figure S3: Gene expression profiling of *Tet1$^{+/+}$, Tet1$^{m/m}$* and *Tet1$^{–/–}$* ESCs and Tet1 genomic occupancy in ESCs.

Figure S4: Genomic occupancy of Ezh2, Sin3a, Chd4 and histone modifications in ESCs.

Figure S5: Analysis of chromatin accessibility in *Tet1$^{+/+}$, Tet1$^{m/m}$* and *Tet1$^{–/–}$* ESCs by ATAC-seq .

Figure S6: Analysis of DNA methylation in ESCs by WGBS.

Figure S7: Comparison of late gestation and adult *Tet1$^{m/m}$* and *Tet1$^{–/–}$* mice .

Table S1: List of oligos used in study

Table S2: List of reagents and resources used in study

Table S3: List of differentially expressed genes (DEGs) with normalized counts, base mean, log2 fold change, p value and p adj value as identified by RNA-seq analysis of Tet1 WT, Tet1 Mut, and Tet1 KO ESCs (comparisons are indicated in each tab headings)

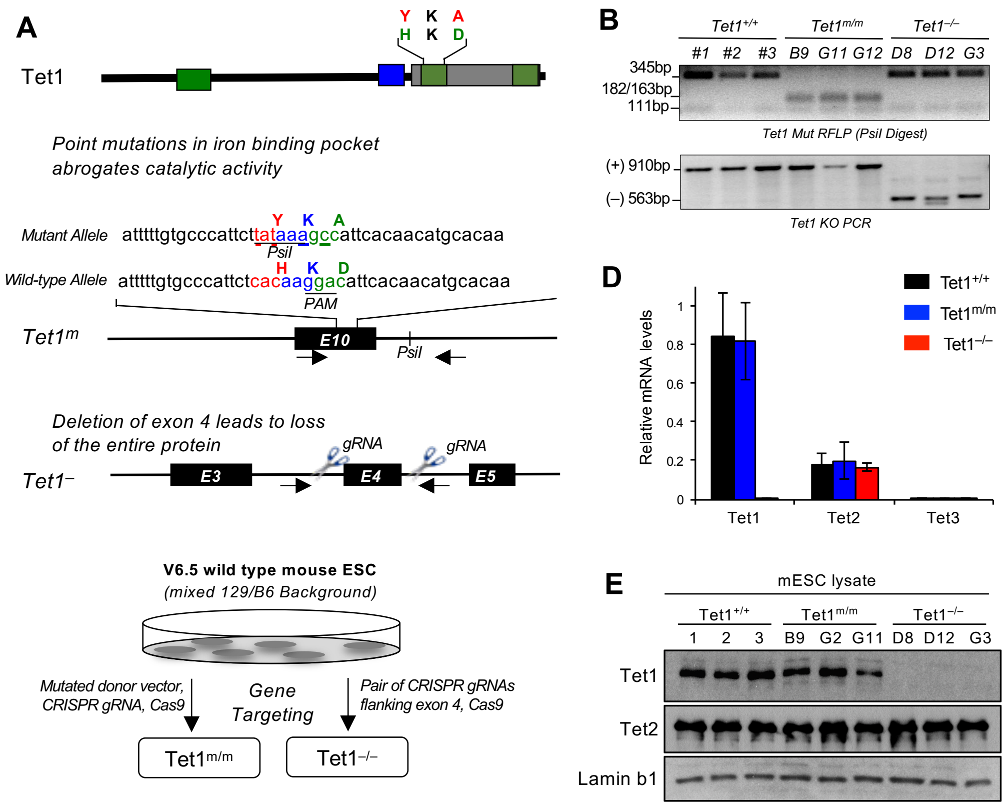

Figure S1. Generation of Tet1 catalytic-mutant (*Tet1*$^{m/m}$) and Tet1-knockout (*Tet1*$^{-/-}$) mouse embryonic stem cells.

A. Schematic of Tet1 protein (top) and gene editing strategy to generate Tet1 catalytic-mutant (*Tet1*$^{m/m}$) and Tet1-knockout (*Tet1*$^{-/-}$) mouse embryonic stem cells (bottom).
B. Confirmation of properly targeted *Tet1*$^{-/-}$ clones by PCR and *Tet1*$^{m/m}$ clones by RFLP (restriction fragment length polymorphism) using PsiI digestion.
C. Confirmation of properly targeted *Tet1*$^{m/m}$ ESC lines by Sanger sequencing.
D. Quantification of *Tet1* (using primers in the deleted exon 4), *Tet2* and *Tet3* mRNA in ESCs of indicated genotypes by RT-qPCR. Normalized to *Gapdh* expression. Error bars represent SEM. n = 3 independent lines of each genotype.
E. Tet1 and Tet2 protein levels in ESCs of the indicated genotypes assessed by Western blot. Lamin b1 was used as a loading control. Note the complete loss of Tet1 in *Tet1*$^{-/-}$ ESCs and normal expression of wild type and catalytic mutant *Tet1* in *Tet1*$^{+/+}$ and *Tet1*$^{m/m}$ ESCs.
F. Quantification of global 5hmC in DNA isolated from ESCs of indicated genotypes by mass spectrometry. n = 3 independent lines of each genotype.

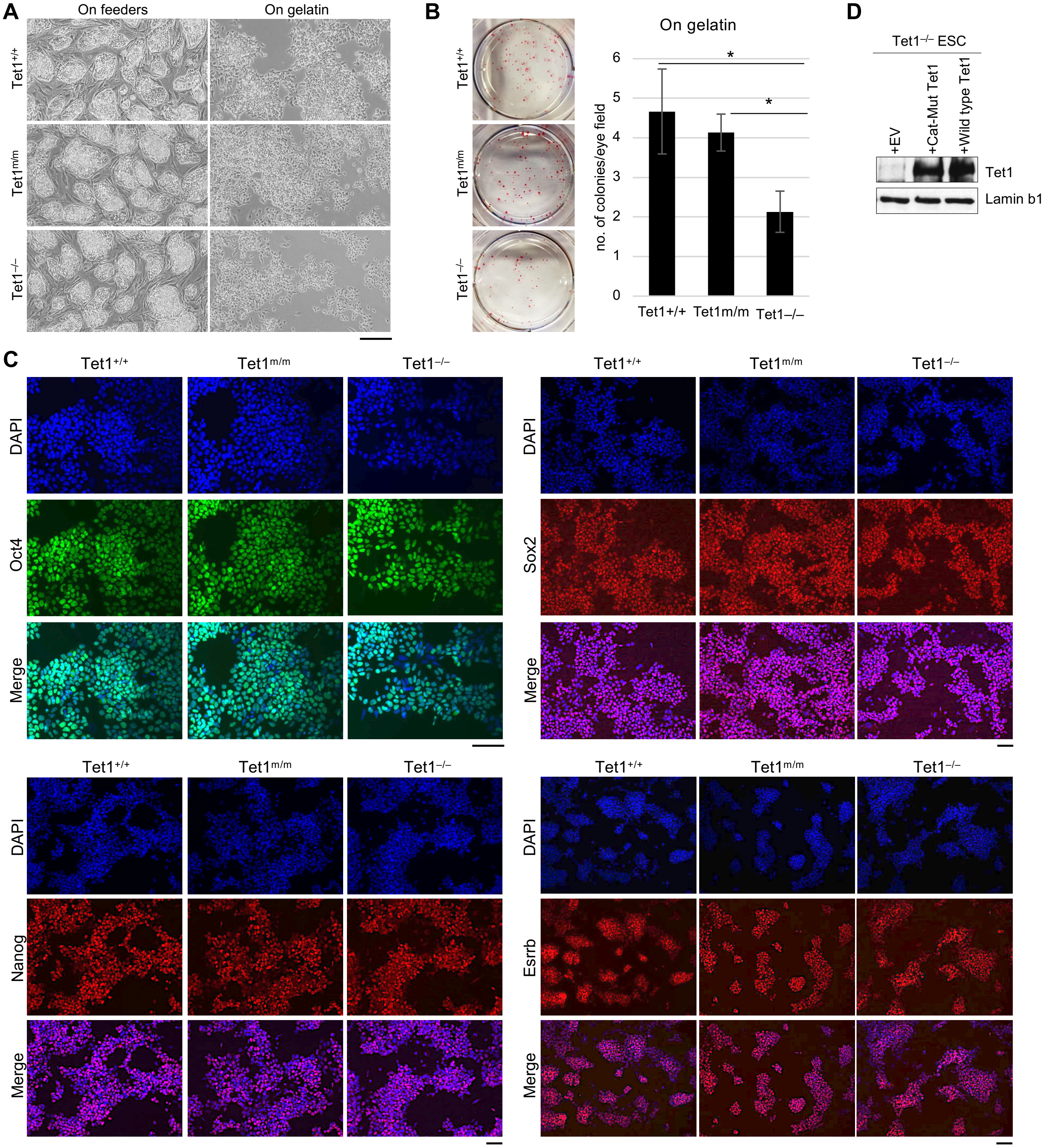

Figure S2. Characterization of Tet1 catalytic-mutant (*Tet1$^{m/m}$*) and Tet1-knockout (*Tet1$^{-/-}$*) mouse ESCs.

(A) Brightfield images of Tet1$^{+/+}$, Tet1$^{m/m}$ and Tet1$^{-/-}$ ESCs grown on feeders or gelatin. Scale bar = 100um

(B) Visualization and quantification of pluripotent colonies by alkaline phosphatase staining in a clonogenicity assay performed on gelatin-coated wells. n = 3 independent lines of each genotype. One-way ANOVA test used.

(C) Immunofluorescence staining of Oct4, Sox2, Nanog and Esrrb in ESCs of the indicated genotypes. DAPI was used to stain nuclei. n = 2 independent lines of each genotype. Scale bar = 100 um

(D) Schematic of re-expression of wild type and catalytic mutant *Tet1* transgenes in *Tet1$^{-/-}$* ESCs (left) and confirmation of expression by Western blot using anti-Tet1 antibody (right). ESCs expressing an empty vector (EV) were used as a control. Lamin b1 was used as a loading control.

A

ESC lines used for RNA-seq	No. of reads from two lanes	After trimming	Alignment rate
Tet1$^{+/+}$_1	38676148	36738470	78.9%
Tet1$^{+/+}$_2	31549406	29918187	79.1%
Tet1$^{m/m}$_1	38719828	36736267	78.9%
Tet1$^{m/m}$_2	37240600	35414057	79.2%
Tet1$^{-/-}$_1	37258361	35441745	79.7%
Tet1$^{-/-}$_2	37378347	35548711	80%

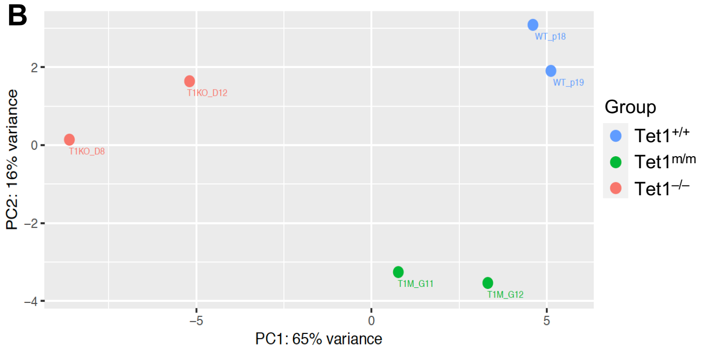

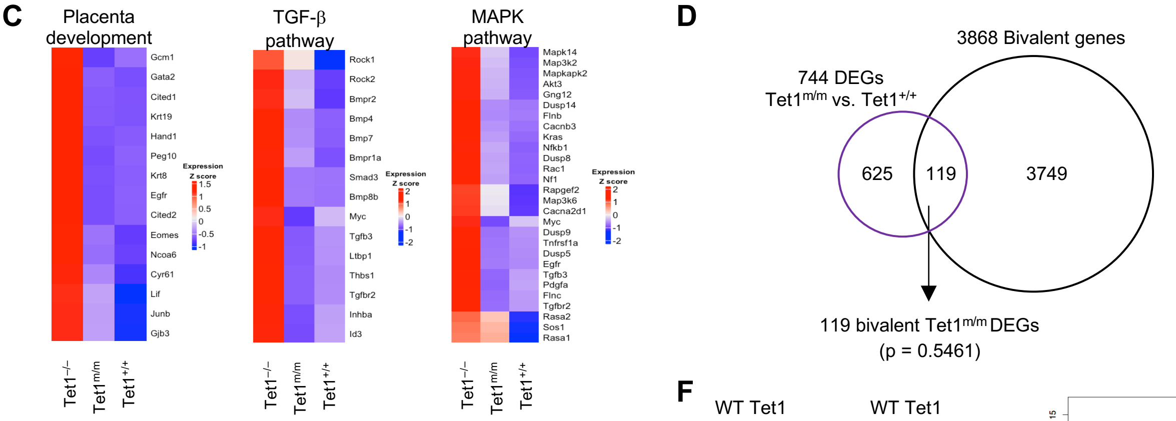

E

Experiment	Samples–Antibody	Clean reads from 75bp paired-end sequencing
Tet1-Flag CUT&Tag	Tet1$^{+/+,Flag/Flag}$ – Flag-M2	20218625
	Tet1$^{m/m,Flag/Flag}$ – Flag-M2	24338219
	Tet1$^{+/+}$ – Flag-M2	15821992
	IgG	5687287
Tet1-GTX CUT&Tag	Tet1$^{+/+}$ – GTX	24096664
	Tet1$^{m/m}$ – GTX	25663303
	Tet1$^{-/-}$ – GTX	23277337
	IgG	6757023

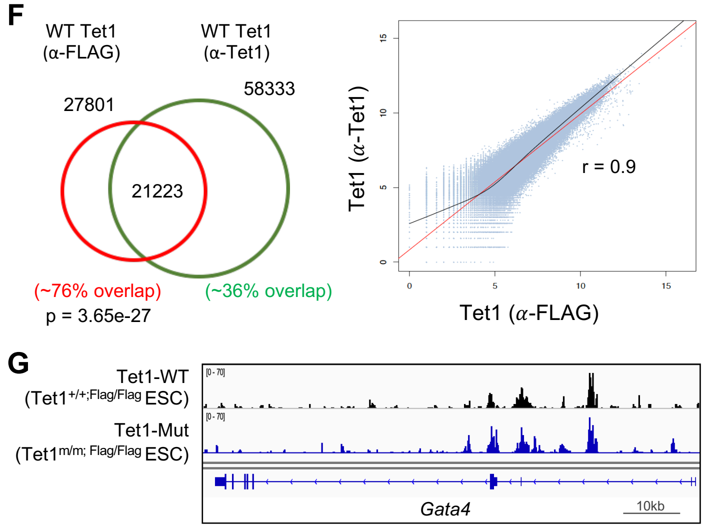

Figure S3. Gene expression profiling of *Tet1$^{+/+}$*, *Tet1$^{m/m}$* and *Tet1$^{-/-}$* ESCs and Tet1 genomic occupancy in ESCs

A. Summary of RNA-seq reads in *Tet1$^{+/+}$*, *Tet1$^{m/m}$* and *Tet1$^{-/-}$* ESCs. n = 2 independent lines of each genotypes.

B. Principal Component Analysis (PCA) plot of genes in each RNA-seq replicate of indicated genotypes.

C. Heatmap of selected differentially expressed genes belonging to three GO terms (placenta development, TGF-β and MAPK pathways) in ESCs. Note the unique upregulation of genes in *Tet1$^{-/-}$* ESCs.

D. Venn diagram showing overlap of 744 DEGs between *Tet1$^{m/m}$* vs. *Tet1$^{+/+}$* DEGs in ESCs and all bivalent genes (3868) in ESCs as identified by Mas *et al*., 2018. Note that the overlap is not statistically significant (p>0.05).

E. Summary of Tet1-Flag and Tet1-GTX (commercial antibody) CUT&Tag data.

F. Venn diagram showing overlap of Tet1 peaks identified by CUT&Tag using anti-Tet1 and anti-FLAG (left). Pearson correlation between anti-Tet1 and anti-FLAG CUT&Tag data (right). Each blue dot represents binding occupancy in 10Kb windows across the entire mouse genome. The black line is the LOWESS smoothing curve and red line is linear regression curve. The correlation coefficient between the two sets was 0.9.

G. Genome browser track snapshot showing enrichment of wild type and catalytic mutant Tet1 CUT&Tag signals at *Gata4* locus in *Tet1$^{+/+, Flag/Flag}$* and *Tet1$^{m/m, Flag/Flag}$* ESCs.

A

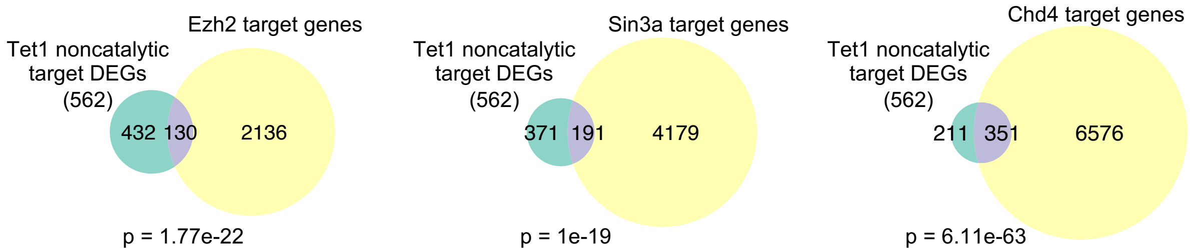

B

Experiment	Samples-Antibody	Clean reads from 75bp paired-end sequencing	Mapped reads before deduplication	Mapped reads after deduplication
Ezh2 CUT&Tag	Tet1$^{+/+}$ – Ezh2	20912826	19784869 (94.61%)	694505
	Tet1$^{m/m}$ – Ezh2	21658497	20852331 (96.28%)	887138
	Tet1$^{-/-}$ – Ezh2	26720333	25750915 (96.37%)	667138
H3K27ac CUT&Tag	Tet1$^{+/+}$ – H3K27ac	10937947	10740559 (98.20%)	2346515
	Tet1$^{m/m}$ – H3K27ac	18488765	18222162 (98.56%)	2807323
	Tet1$^{-/-}$ – H3K27ac	18570702	18260184 (98.33%)	3138965
Ezh2 and H3K27ac CUT&Tag	IgG	10142676	8054521 (79.41%)	170654
Sin3a CUT&Tag	Tet1$^{+/+}$ – Sin3a	40206138	39703678 (98.75%)	4924433
	Tet1$^{m/m}$ – Sin3a	43057911	42516333 (98.74%)	5710748
	Tet1$^{-/-}$ – Sin3a	39013277	38454430 (98.57%)	3557727
	IgG	32597893	32050026 (98.32%)	1335794
H3K4me3 CUT&Tag	Tet1$^{+/+}$ – H3K4me3	24267636	24022538 (98.99%)	14748213
	Tet1$^{m/m}$ – H3K4me3	20571982	20371812 (99.03%)	12793322
	Tet1$^{-/-}$ – H3K4me3	19305817	19110736 (98.99%)	12641866
H3K27me3 CUT&Tag	Tet1$^{+/+}$ – H3K27me3	19926906	19749648 (99.11%)	16696005
	Tet1$^{m/m}$ – H3K27me3	25997795	25742166 (99.02%)	21451481
	Tet1$^{-/-}$ – H3K27me3	15561864	15424307 (99.12%)	13431875
H3K4me3 and H3K27me3 CUT&Tag	IgG	8612127	8372394 (97.22%)	343708

Experiment	Samples-Antibody	Clean reads from 35bp paired-end sequencing	Mapped reads with fragments ≤ 120bp	Deduplicated mapped reads with fragments ≤ 120bp
Chd4 CUT&RUN	Tet1$^{+/+}$ – Chd4	56575175	37104354 (65.58%)	31696239
	Tet1$^{m/m}$ – Chd4	28553493	19708083 (69.02%)	17046002
	Tet1$^{-/-}$ – Chd4	33375263	21924972 (65.69%)	18951978
	IgG	53426005	37214695 (69.66%)	34548106

Figure S4. Genomic occupancy of Ezh2, Sin3a, Chd4 and histone modifications in ESCs.

A. Venn diagrams showing overlap between Tet1 noncatalytic target genes (genes bound by Tet1 at promoters and deregulated in *Tet1*$^{-/-}$ ESC only) and Ezh2, Sin3a and Chd4 target genes (genes bound by any of these factors at promoters based on published ChIP-seq datasets, see methods). p-values calculated by a hypergeometric test denote significance of overlap using all mouse genes as the background.

B. Summary of sequencing read counts of Ezh2, Sin3a, H3K27ac, H3K4me3, H3K27me3 CUT&Tag data, and Chd4 CUT&RUN data.

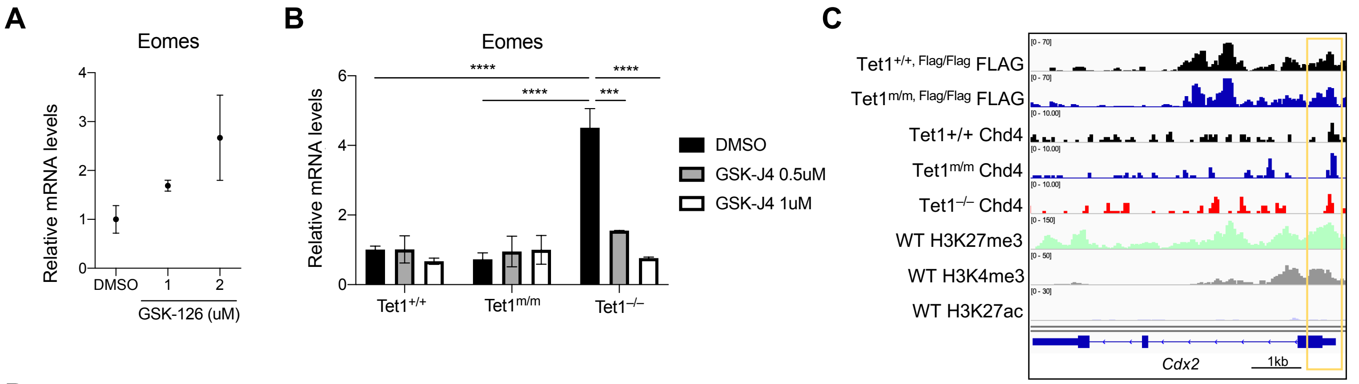

D

Sample Name	Untrimmed reads	Trimmed reads	Mapped reads	Overall alignment rate	Reads after filtering mitochondria and duplicates	MACS2 peaks after filtering blacklist	Differentially accessible regions (DARs) compared to Tet1$^{+/+}$
1-Tet1$^{+/+}$	57393543	57198537	55990585	97.89%	50462056		
2-Tet1$^{+/+}$	36211441	36091979	35416545	98.13%	32329465	109851	N.A.
3-Tet1$^{+/+}$	44823215	44674969	44143072	98.81%	41137700		
1-Tet1$^{m/m}$	46790094	46614471	45465565	97.54%	40009263		
2-Tet1$^{m/m}$	44889847	44743380	44071148	98.50%	40332240	146337	**28 DARs** 12 gained 16 lost
3-Tet1$^{m/m}$	31732306	31624457	31222090	98.73%	29392838		
1-Tet1$^{-/-}$	33707406	33596127	33215057	98.87%	30825248		
2-Tet1$^{-/-}$	51724933	51555533	50914795	98.76%	46734662	122618	**658 DARs** 320 gained 338 lost
3-Tet1$^{-/-}$	37401081	37273638	36856080	98.88%	34162359		

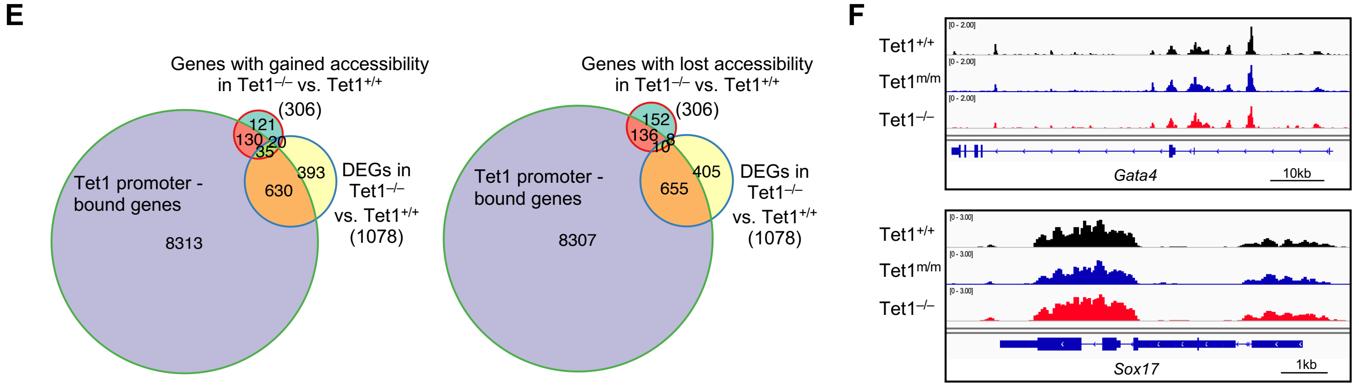

Figure S5. Analysis of chromatin accessibility in *Tet1*$^{+/+}$, *Tet1*$^{m/m}$ and *Tet1*$^{-/-}$ ESCs by ATAC-seq.

A. mRNA levels of *Eomes* quantified by RT-qPCR in *Tet1*$^{+/+}$ ESCs treated with Ezh2 inhibitor GSK-126 and DMSO control. Data normalized to *Gapdh*. n = 3 independent lines of each genotype.

B. mRNA levels of *Eomes* quantified by RT-qPCR in *Tet1*$^{+/+}$, *Tet1*$^{m/m}$ and *Tet1*$^{-/-}$ ESCs treated with histone demethylase inhibitor GSK-J4 and DMSO control. Data normalized to *Gapdh*. n = 3 independent lines of each genotype. One-way ANOVA test used.

C. Representative genome browser tracks depicting Chd4 enrichment at the promoter of the bivalent gene *Cdx2* in *Tet1*$^{+/+}$, *Tet1*$^{m/m}$ and *Tet1*$^{-/-}$ ESCs. Tet1 and histone mark enrichments are also shown.

D. Summary of ATAC-seq data and read numbers in *Tet1*$^{+/+}$, *Tet1*$^{m/m}$ and *Tet1*$^{-/-}$ ESCs.

E. Venn diagrams showing overlap between genes uniquely deregulated in *Tet1*$^{-/-}$ ESCs, Tet1 promoter-bound genes and genes with gained or lost accessibility as assessed by ATAC-seq.

F. Representative genome browser tracks showing ATAC-seq peaks at selected bivalent genes in *Tet1*$^{+/+}$, *Tet1*$^{m/m}$ and *Tet1*$^{-/-}$ ESCs. Note no change in accessibility at bivalent genes upon Tet1 loss.

A

Sample name	Clean Reads	% duplications	% Aligned after deduplication	non-CpG context C to T conversion rate (%)	Lambda DNA conversion efficiency (C to T rate, Cs are from CpG, CHG, CHH) (%)
1-Tet1$^{+/+}$	1,095,248,314	20.1%	79.9%	98.91	99.39
2-Tet1$^{+/+}$	1,110,558,298	21.4%	78.6%	98.92	99.42
3-Tet1$^{m/m}$	1,092,528,326	22.2%	77.8%	98.95	99.43
4-Tet1$^{m/m}$	1,095,008,860	16.2%	83.8%	99.00	99.46
5-Tet1$^{-/-}$	1,114,003,694	21.2%	78.8%	99.13	99.47
6-Tet1$^{-/-}$	1,093,327,318	20.0%	80.0%	99.02	99.45

Sample Name	Total no. of Cs analyzed, in millions	Depth of C	% methylated C (Cs from CpG, CHG, CHH)	Methylated C's in CpG context	Unmethylated C's in CpG context	% mCpG	% mCHG	% mCHH	Total symmetric CpGs	Methylated symmetric CpGs
1-Tet1$^{+/+}$	11289.2	10.21x	3.06	345,908,754	141,946,328	70.9%	1.2%	1.0%	21,775,389	19,392,968 (89.1%)
2-Tet1$^{+/+}$	11962.3	10.82x	3.06	365,674,087	162,266,708	69.3%	1.2%	1.0%	21,773,040	19,431,563 (89.2%)
3-Tet1$^{m/m}$	12126.7	10.97x	3.25	394,094,888	148,976,636	72.6%	1.2%	1.0%	21,775,901	19,495,502 (89.5%)
4-Tet1$^{m/m}$	12454.7	11.27x	3.22	400,504,042	147,335,817	73.1%	1.2%	1.0%	21,774,102	19,457,162 (89.4%)
5-Tet1$^{-/-}$	11735.1	10.62x	3.46	406,306,948	132,789,512	75.4%	0.9%	0.8%	21,781,529	19,476,026 (89.4%)
6-Tet1$^{-/-}$	11105.6	10.05x	3.01	334,728,081	160,154,452	67.6%	1.1%	0.9%	21,768,653	19,444,166 (89.3%)

Data was mapped to mouse genome mm10 by bismark methylpy pipeline   Genome wide Cs: 1105.3 million, Average coverage > 10x at each cytosine site

B Differentially Methylated Regions (DMRs) by BSmooth

	Total DMR	Hyper DMR	Hypo DMR	Common DMRs
Tet1$^{-/-}$ vs. Tet1$^{+/+}$	22094	21042	1052	12742<br>- 12631hyper<br>- 111 hypo
Tet1$^{m/m}$ vs. Tet1$^{+/+}$	36968	36484	484	

C

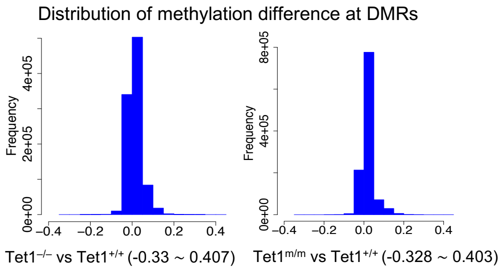

D

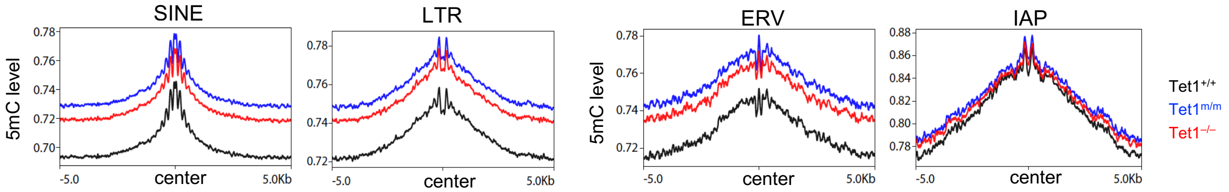

E

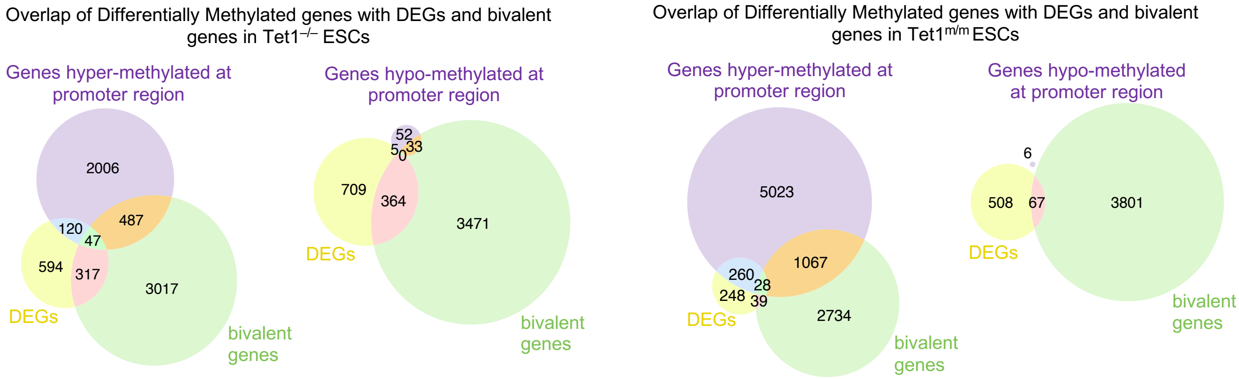

Figure S6. Analysis of DNA methylation in ESCs by WGBS

A. Summary of WGBS data and read counts.

B. Differentially Methylated Regions (DMRs) in *Tet1$^{-/-}$ vs. Tet1$^{+/+}$* ESCs and *Tet1$^{m/m}$ vs. Tet1$^{+/+}$* ESCs.

C. Distribution of methylation difference at DMRs. Most DMRs are hypermethylated with ~10% change in methylation.

D. 5mC levels at SINE (Short interspersed nuclear element), LTR (Long terminal repeats), ERV (Endogenous Retrovirus elements) and IAP (Intracisternal A-type particle) in ESC. All 48920 hyper DMRs used in analysis.

E. Overlap of differentially methylated genes, differentially expressed genes, and bivalent genes. No correlation between bivalent gene deregulation and methylation upon loss of Tet1 or its catalytic activity.

A

Mendelian ratio of E11.5 Tet1$^{m/m}$ and Tet1$^{-/-}$ embryos

+/m x +/m<br>Total of 5 litters<br>Avg. Litter size = 6.8	+/+	+/m	m/m	Ratio
	11 (32.3%)	14 (41.2%)	9 (26.5%)	1:2:1
+/– x +/–<br>Total of 5 litters<br>Avg. Litter size = 7.8	+/+	+/–	–/–	Ratio
	12 (30.8%)	17 (43.6%)	10 (25.6%)	1:2:1

B

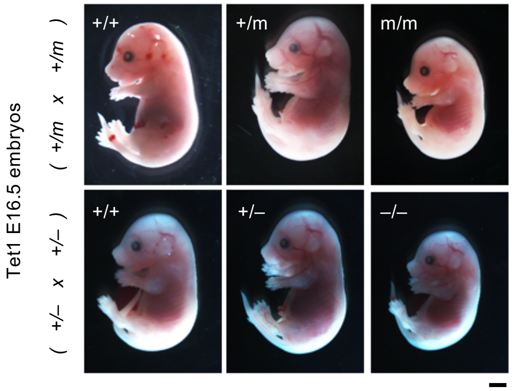

C

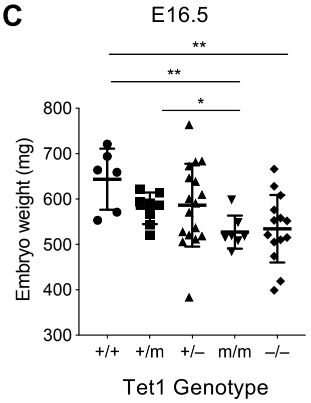

D

Mendelian ratio of E16.5 Tet1$^{m/m}$ and Tet1$^{-/-}$ embryos

+/m x +/m<br>Total of 8 litters<br>Avg. Litter size = 6.5	+/+	+/m	m/m	Ratio
	12 (23.1%)	27 (51.9%)	13 (25.0%)	1:2:1
+/– x +/–<br>Total of 6 litters<br>Avg. Litter size = 7	+/+	+/–	–/–	Ratio
	6 (11.5%)	22 (52.4%)	14 (30.0%)	1:2:1

E

Mendelian ratio of weaned Tet1$^{m/m}$ and Tet1$^{-/-}$ mice

+/m x +/m<br>Total of 19 litters<br>Avg. Litter size = 7.2	+/+	+/m	m/m	Ratio
	43 (31.4%)	71 (51.8%)	23 (16.8%)	2:3:1
+/– x +/–<br>Total of 13 litters<br>Avg. Litter size = 5.2	+/+	+/–	–/–	Ratio
	18 (26.5%)	38 (55.9%)	12 (17.6%)	2:3:1

F

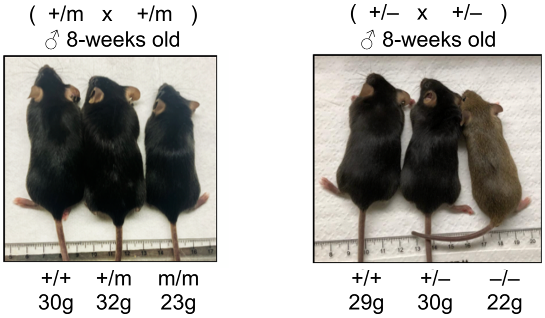

Figure S7. Comparison of late gestation and adult *Tet1$^{m/m}$* and *Tet1$^{-/-}$* mice.

A. Summary of litter size and Mendelian ratio of *Tet1$^{m/m}$* and *Tet1$^{-/-}$* E11.5 embryos generated from intercrosses of respective heterozygote mice.

B. Gross images of E16.5 embryos of the indicated genotypes. Scale bar = 2mm.

C. Weight of E16.5 embryos of the indicated genotypes. One-way ANOVA test used. Statistically significant (*p<0.05, **p<0.01)

D. Summary of litter size and Mendelian ratio of *Tet1$^{m/m}$* and *Tet1$^{-/-}$* E16.5 embryos generated from intercrosses of heterozygote mice.

E. Summary of litter size and Mendelian ratio of *Tet1$^{m/m}$* and *Tet1$^{-/-}$* weaned mice generated from intercrosses of respective heterozygote mice.

F. Gross images of male Tet1 mice of indicated genotypes.

Table S1: List of oligos used in study

Name	Sequence (5'-3')	Purpose	Source
Tet1 RT-qPCR For	GCTGGATTGAAGGAAGAGGA	Real time qPCR	Dawlaty et al. Cell Stem Cell 2011
Tet1 RT-qPCR Rev	GTCTCCATGAGCTCCCTGAC	Real time qPCR	Dawlaty et al. Cell Stem Cell 2011
Tet2 RT-qPCR For	GTCAACAGGACATGATCCAGGAG	Real time qPCR	Zhe et al., Blood 2011
Tet2 RT-qPCR Rev	CCTGTTCCATCAGGCTTGCT	Real time qPCR	Zhe et al., Blood 2011
Tet3 RT-qPCR For	TCCGGATTGAGAAGGTCATC	Real time qPCR	Dawlaty et al. Developmental Cell 2014
Tet3 RT-qPCR Rev	CCAGGCCAGGATCAAGATAA	Real time qPCR	Dawlaty et al. Developmental Cell 2014
Nanog RT-qPCR For	AAGCAGAAGATGCGGACTGT	Real time qPCR	Dawlaty et al. Developmental Cell 2014
Nanog RT-qPCR Rev	ATCTGCTGGGAGGCTGAGGTA	Real time qPCR	Dawlaty et al. Developmental Cell 2014
Pou5f1 RT-qPCR For	ACATCGCCAATCAGCTTGG	Real time qPCR	Dawlaty et al. Developmental Cell 2014
Pou5f1 RT-qPCR Rev	AGAACCATACTCGAACCACATCC	Real time qPCR	Dawlaty et al. Developmental Cell 2014
Klf4 RT-qPCR For	GAAGACCAGGATTCCCTTGA	Real time qPCR	Dawlaty et al. Developmental Cell 2014
Klf4 RT-qPCR Rev	CCAAGCACCATCATTTAGGC	Real time qPCR	Dawlaty et al. Developmental Cell 2014
Sox17 RT-qPCR For	GATGCGGGATACGCCAGTG	Real time qPCR	Dawlaty et al. Developmental Cell 2014
Sox17 RT-qPCR Rev	CCACCACCTCGCCTTTCAC	Real time qPCR	Dawlaty et al. Developmental Cell 2014
Gata4 RT-qPCR For	CAGAAGGCAGAGAGTGTGTC	Real time qPCR	Ravichandran et al. Cell Reports 2019
Gata4 RT-qPCR Rev	AGTGGCATTGCTGGAGTTAC	Real time qPCR	Ravichandran et al. Cell Reports 2019
Gata6 RT-qPCR For	GAGCTGGTGCTACCAAGAGG	Real time qPCR	Ito et al. Nature 2011
Gata6 RT-qPCR Rev	TGCAAAAGCCCATCTCTTCT	Real time qPCR	Ito et al. Nature 2011
FoxA2 RT-qPCR For	CATCCGACTGGAGCAGCTACTA	Real time qPCR	Gonzales-Cope et al BMC Genomic 2016
FoxA2 RT-qPCR Rev	AGCGCCCACATAGGATGACAT	Real time qPCR	Gonzales-Cope et al BMC Genomic 2016
Bin1 RT-qPCR For	CAAGGCAAACTACAGGCTCATC	Real time qPCR	Vella et al. Molecular Cell 2013
Bin1 RT-qPCR Rev	CCACGTTCATCTCCTCGAAC	Real time qPCR	Vella et al. Molecular Cell 2013
Pax6 RT-qPCR For	AACAACCTGCCTATGCAACC	Real time qPCR	Dawlaty et al. Cell Stem Cell 2011
Pax6 RT-qPCR Rev	ACTTGGACGGGAACTGACAC	Real time qPCR	Dawlaty et al. Cell Stem Cell 2011
Otx2 RT-qPCR For	GACGTTCTGGAAGCTCTGTT	Real time qPCR	This paper
Otx2 RT-qPCR Rev	ATGGTTGGGACTGAGGTACT	Real time qPCR	This paper
Nr4a2 RT-qPCR For	GTGTTCAGGCGCAGTATGG	Real time qPCR	Li et al iScience 2019
Nr4a2 RT-qPCR Rev	TGGCAGTAATTTCAGTGTTGGT	Real time qPCR	Li et al iScience 2019
Dab1 RT-qPCR For	GCACAAAAGGATAAGCAGTGTGA	Real time qPCR	Vella et al. Molecular Cell 2013
Dab1 RT-qPCR Rev	AACCCTGACGTGGTTCAGAAA	Real time qPCR	Vella et al. Molecular Cell 2013
Fgf5 RT-qPCR For	ATGAGCCTGTCCTTGCTCTT	Real time qPCR	Poh et al. Nature Communications 2014
Fgf5 RT-qPCR Rev	TCGTGGGAGCCATTGACTTT	Real time qPCR	Poh et al. Nature Communications 2014
Eomes RT-qPCR For	TGCAAGAGAAAGCGCCTGTCTC	Real time qPCR	Koh et al Cell Stem Cell 2011
Eomes RT-qPCR Rev	CAATCCAGCACCTTGAACGACC	Real time qPCR	Koh et al Cell Stem Cell 2011
Cdx2 RT-qPCR For	CGAGCCCTTGAGTCCTGTGA	Real time qPCR	Gu et al. Stem Cell Reports 2018
Cdx2 RT-qPCR Rev	AACCCCAGGGACAGAACC	Real time qPCR	Gu et al. Stem Cell Reports 2018
Egfr RT-qPCR For	GCCATCTGGGCCAAAGATACC	Real time qPCR	Liu et al. Stem Cell Research & Therapy 2016
Egfr RT-qPCR Rev	GTCTTCGCATGAATAGGCCAAT	Real time qPCR	Liu et al. Stem Cell Research & Therapy 2016
Krt7 RT-qPCR For	AGGAGATCAACCGACGCAC	Real time qPCR	Liu et al. Stem Cell Research & Therapy 2016
Krt7 RT-qPCR Rev	GTCTCGTGAAGGGTCTTGAGG	Real time qPCR	Liu et al. Stem Cell Research & Therapy 2016
Gapdh RT-qPCR For	GTGTTCCTACCCCCAATGTGT	Real time qPCR	Dawlaty et al. Cell Stem Cell 2011
Gapdh RT-qPCR Rev	ATTGTCATACCAGGAAATGAGCTT	Real time qPCR	Dawlaty et al. Cell Stem Cell 2011
Cdx2 For	CCAGGTTGGAAGGAGGAAGC	ChIP-qPCR	Rugg-Gunn et al PNAS 2010
Cdx2 Rev	ACCACCCCCAGAAACACGAT	ChIP-qPCR	Rugg-Gunn et al PNAS 2010
FoxA2 For	TCCTCCTGAAGTCATCCCACAA	ChIP-qPCR	Rugg-Gunn et al PNAS 2010
FoxA2 Rev	TAAATCCAAGGTGCCCAAAGC	ChIP-qPCR	Rugg-Gunn et al PNAS 2010
Gata6 For	TTTTTCTGGAGCTCGCGTTGGATATGA	ChIP-qPCR	Rugg-Gunn et al PNAS 2010
Gatat6 Rev	GAGTTCCGCACGTGGAAATAGCTAGAA	ChIP-qPCR	Rugg-Gunn et al PNAS 2010
Sox17 For	CACCAACCCGCTTGCTACAG	ChIP-qPCR	Rugg-Gunn et al PNAS 2010
Sox17 Rev	TAAGCCACATCCCCAAAGCA	ChIP-qPCR	Rugg-Gunn et al PNAS 2010

Table S1: List of oligos used in study (Continued)

Name	Sequence (5'-3')	Purpose	Source
Tet1 KO Genotyping For	AACTGATTCCCTTCGTGCAG	PCR Genotyping KO Mice and ESCs	This paper
Tet1 KO Genotyping Rev	TTAAAGCATGGGTGGGAGTC	PCR Genotyping KO Mice and ESC	This paper
Tet1 Mut Genotyping For	CATATGCAGACAAAACCACAC	PCR Genotyping Mut Mice and ESC	This paper
Tet1 Mut Genotyping Rev	TGTTCCTTCTGCAGCATTGT	PCR Genotyping Mut Mice and ESC	This paper
Rosa26-14930	TCTTGCGAACCTCATCACTC	PCR Genotyping of Rosa26-CreER mice	Jax Lab
Rosa26-0IMR9020	AAGGGAGCTGCAGTGGAGTA	PCR Genotyping of Rosa26-CreER mice	Jax Lab
Rosa26-0IMR9021	CCGAAAATCTGTGGGAAGTC	PCR Genotyping of Rosa26-CreER mice	Jax Lab
Tet1 Conditional For	AACTGATTCCCTTCGTGCAG	PCR Genotyping Conditional Tet1 allele	This paper
Tet1 Conditional Rev	TTAAAGCATGGGTGGGAGTC	PCR Genotyping Conditional Tet1 allele	This paper
FLAG gRNA For	CACCGTGCGGGACCCTACAATCGTT	Targeting 3XFlag tag into the stop codon of Tet1	Gu et al Genome Biol. 2018
FLAG gRNA Rev	AAACAACGATTGTAGGGTCCCGCAC	Targeting 3XFlag tag into the stop codon of Tet1	Gu et al Genome Biol. 2018
3x FLAG genotyping For	TGATGTATCCCCCGAAGCCA	Genotyping 3x Flag allele	Gu et al Genome Biol. 2018
3x FLAG genotyping Rev	CGGAGTTGAAATGGGCGAAAC	Genotyping 3x Flag allele	Gu et al Genome Biol. 2018
3X FLAG ssDNA	CAATGTTGTTACCGTGTCCCCATACTCTCTCACTCATGTT GCGGGACCCTACAATAGATGGGTCGGCGGCAGTGGAGACT ACAAAGACCATGACGGTGATTATAAAGATCATGACATCGATT ACAAGGATGACGATGACAAGTAAAGGCTTCTCTCATGTAAT GCCTTTGCTAATGTGGTGTAGTGGGTatttttgtttg	Targeting 3XFlag tag into the stop codon of Tet1	Gu et al Genome Biol. 2018
Tet1 exon 4 Left gRNA For	CACCAGGGGCTGGCATTGGACTTC	Deleting Tet1 Exon 4	This paper
Tet1 exon 4 Left gRNA Rev	AAACGAAGTCCAATGCCAGCCCCT	Deleting Tet1 Exon 4	This paper
Tet1 exon 4 Right gRNA For	CACCCAGTCCTCCTGAGTCCTCCC	Deleting Tet1 Exon 4	This paper
Tet1 exon 4 Right gRNA Rev	AAACGGGAGGACTCAGGAGGACTG	Deleting Tet1 Exon 4	This paper
Tet1 exon 10 gRNA For	CACCGTTTTTGTGCCCATTCTCACA	Mutating Tet1 Exon 10	This paper
Tet1 exon 10 gRNA Rev	AAACTGTGAGAATGGGCACAAAAAC	Mutating Tet1 Exon 10	This paper
Tet1 mutant gene block sequence	acagacagacaggggctac(…700bp…)tcttattttacagttttgacatttatttctaag GTGGAATATGAAGAAGTTGCTGGAGACTGTCGACTTGGAAA TGAAGAGGGGCGTCCTTTCTCTGGTGTCACCTGTTGCATGG ATTTTTGTGCCCATTCTTATAAAGCCATTCACAACATGCACA ACGGAAGCACCGTGgtatgcact (….570bp…) agaacaggccttattcctctcctgtccctc	Introducing HKD to YKA mutation in catalytic domain of Tet1 in exon 10	This paper

TABLE S2: List of reagents and resources used in study

REAGENT or RESOURCE	SOURCE	IDENTIFIER
**Antibodies**		
Anti-Tet1	GeneTex	GTX125888
Anti-Tet2	Abcam	ab124297
Anti-Ezh2	Cell Signaling Technology	5246
Anti-Suz12	Cell Signaling Technology	3737
Anti-Sin3a	Abcam	ab3479
Anti-H3K27me3	Cell Signaling Technology	9733
Anti-H3K4me3	Active Motif	39060
Anti-H3K27ac	Abcam	ab4729
Anti-Chd4	Cell Signaling Technology	12011
Anti-Lamin b1	ABclonal	A1910
Anti-Gata6	R&D	AF1700
Goat anti-Rabbit IgG-HRP	CalBiochem	401393
Goat anti-Mouse IgG-HRP	CalBiochem	401253
Alexa Flour 568 anti-Goat	Thermo Fisher	A11057
Rabbit (DA1E) IgG XP Isotype Control	Cell Signaling Technology	3900
Guinea pig anti-Rabbit IgG	Antibodies Online	ABIN101961
**Critical Commercial Assays**		
E.Z.N.A. Total RNA kit	Omega	R6834-02
Superscript III first strand	Invitrogen	18080-400
Qubit dsDNA HS assay kit	Invitrogen	Q32851
Xfect mESC polymer	Clonetech	631320
XtremeGene 9 DNA transfection reagent	Roche	06365787001
Quick-DNA Miniprep Kit	Zymo Research	D3024
SMART-Seq v4 Ultra Low Input RNA kit	Takara Bio	634895
NEBNext Ultra II DNA Library Prep kit Illumina	New England Biolabs	E7645
**Deposited Data**		
RNA-seq, CUT&Tag-seq, CUT&RUN-seq, WGBS, and ATAC-seq datasets	This paper	GEO: GSE176389
**Experimental Models: Cell Lines and Mice**		
V6.5 Tet1 $^{+/+}$ mESC	This paper	N.A.
V6.5 Tet1 $^{m/m}$ mESC	This paper	N.A.
V6.5 Tet1 $^{-/-}$ mESC	This paper	N.A.
SCID mice	Taconic	Model#ICRSC-M
129/B6 Tet1 $^{m/m}$ mice	This paper	N.A.
129/B6 Tet1 $^{-/-}$ mice	Dawlaty et al Cell Stem Cell 2011	N.A.
129/B6 Tet1 $^{f/f}$ *Rosa26-CreER* mice	This paper	N.A.
**Recombinant DNA**		
pX330-GFP-gRNA Tet1 Exon 4 right	This paper	N.A.
pX330-GFP-gRNA Tet1 Exon 4 left	This paper	N.A.
pX330-GFP-gRNA Tet1 Exon 10 mut	This paper	N.A.
Tet1 mutant donor vector	This paper	N.A.
PiggyBac-EV-hygro	Dawlaty et al Developmental Cell 2014	N.A.
PiggyBac-mTet1-WT-hygro	Dawlaty et al Developmental Cell 2014	N.A.
PiggyBac-mTet1-Mut-hygro	This paper	N.A.
FUW-GFP	Dawlaty et al Developmental Cell 2014	N.A.

TABLE S2: List of reagents and resources used in study (Continued)

REAGENT or RESOURCE	SOURCE
**Software and Algorithms**	
FastQC v0.11.4	https://www.bioinformatics.babraham.ac.uk/projects/fastqc/
Trim galore v0.4.1	https://github.com/FelixKrueger/TrimGalore
MACS2 v2.1.0	https://github.com/taoliu/MACS/
Bowtie v2.2.3	http://bowtie-bio.sourceforge.net/bowtie2/index.shtml
Tophat v2.0.13	https://ccb.jhu.edu/software/tophat/index.shtml
HTSeq v0.6.1	https://github.com/simon-anders/htseq
Cufflinks v2.2	http://cole-trapnell-lab.github.io/cufflinks/
DESeq2 v1.20.0	http://www.bioconductor.org/packages/release/bioc/html/DESeq2.html
DAVID 6.8	https://david.ncifcrf.gov/
Picard v2.3.0	https://broadinstitute.github.io/picard/
seqMINER v1.2.1	http://bips.u-strasbg.fr/
deepTools v3.1.0	https://github.com/deeptools/deepTools
ChIPseeker v1.16.1	https://bioconductor.org/packages/release/bioc/html/ChIPseeker.html
SAMtools v1.9	https://sourceforge.net/projects/samtools/files/samtools/1.9/
bedtools2 v2.26.0	https://github.com/arq5x/bedtools2/releases
SOAPnuke v1.5.5	https://github.com/BGI-flexlab/SOAPnuke
Bismark v0.18.1	https://www.bioinformatics.babraham.ac.uk/projects/bismark/
Methylpy v1.4.0	https://github.com/yupenghe/methylpy
methimpute v1.8.0	https://www.bioconductor.org/packages/release/bioc/html/methimpute.html
MethPipe v 3.4.3	http://smithlabresearch.org/software/methpipe/
bsseq v1.16.1	http://bioconductor.org/packages/release/bioc/html/bsseq.html
HOMER v4.7	http://homer.ucsd.edu/homer/
Integrative Genomics Viewer (IGV) v2.5.0	http://software.broadinstitute.org/software/igv/
ImageJ	https://imagej.nih.gov/ij/
FlowJo v10.4	https://www.flowjo.com/solutions/flowjo
GraphPad Prism 7	https://www.graphpad.com/
